# Altered Muscle Contributions are Required to Support the Stance Limb During Voluntary Toe-Walking

**DOI:** 10.3389/fbioe.2022.810560

**Published:** 2022-04-11

**Authors:** Enrico De Pieri, Jacqueline Romkes, Christian Wyss, Reinald Brunner, Elke Viehweger

**Affiliations:** ^1^ Laboratory for Movement Analysis, University of Basel Children’s Hospital, Basel, Switzerland; ^2^ Department of Biomedical Engineering, University of Basel, Basel, Switzerland; ^3^ Department of Paediatric Orthopaedics, University of Basel Children’s Hospital, Basel, Switzerland

**Keywords:** toe-walking, equinus gait, musculoskeletal modeling, muscle function, muscle contributions, joint moments, support moment

## Abstract

Toe-walking characterizes several neuromuscular conditions and is associated with a reduction in gait stability and efficiency, as well as in life quality. The optimal choice of treatment depends on a correct understanding of the underlying pathology and on the individual biomechanics of walking. The objective of this study was to describe gait deviations occurring in a cohort of healthy adult subjects when mimicking a unilateral toe-walking pattern compared to their normal heel-to-toe gait pattern. The focus was to characterize the functional adaptations of the major lower-limb muscles which are required in order to toe walk. Musculoskeletal modeling was used to estimate the required muscle contributions to the joint sagittal moments. The support moment, defined as the sum of the sagittal extensive moments at the ankle, knee, and hip joints, was used to evaluate the overall muscular effort necessary to maintain stance limb stability and prevent the collapse of the knee. Compared to a normal heel-to-toe gait pattern, toe-walking was characterized by significantly different lower-limb kinematics and kinetics. The altered kinetic demands at each joint translated into different necessary moment contributions from most muscles. In particular, an earlier and prolonged ankle plantarflexion contribution was required from the soleus and gastrocnemius during most of the stance phase. The hip extensors had to provide a higher extensive moment during loading response, while a significantly higher knee extension contribution from the vasti was necessary during mid-stance. Compensatory muscular activations are therefore functionally required at every joint level in order to toe walk. A higher support moment during toe-walking indicates an overall higher muscular effort necessary to maintain stance limb stability and prevent the collapse of the knee. Higher muscular demands during gait may lead to fatigue, pain, and reduced quality of life. Toe-walking is indeed associated with significantly larger muscle forces exerted by the quadriceps to the patella and prolonged force transmission through the Achilles tendon during stance phase. Optimal treatment options should therefore account for muscular demands and potential overloads associated with specific compensatory mechanisms.

## Introduction

Heel-striking is considered a vital feature of a normal gait pattern. The gait of most children reaches a fairly mature pattern approximately one year after the initiation of walking (Bertsch et al., 2004). During the first year of bipedal walking, typically developing children learn to heel-strike, allowing them to walk more efficiently and stabilize their motion (Hallemans et al., 2006; Zeininger et al., 2018). In some children, however, a heel-strike pattern is either not developed at all or lost during the maturation of gait. These children may remain on forefoot during all phases of the gait cycle, or the heel may touch the ground only at a later point later during the step. These forefoot or flatfoot patterns are referred to as toe-walking. Toe-walking is observed in children diagnosed with cerebral palsy (CP), autism, and various other neurologic and orthopedic pathologies (Schweizer et al., 2013). In the absence of neurological, orthopedic or psychiatric diseases, it is referred to as idiopathic toe-walking (ITW) (Engelbert et al., 2011). In patients affected by CP, alterations of the gait pattern persist until adulthood, often determining a decline in mobility (Morgan and McGinley, 2013). Toe-walking is also observed in adults who suffered a stroke (Balaban and Tok, 2014) or a traumatic brain injury (TBI) (Williams et al., 2009). Both stroke and TBI patients can present equinus foot deformities which are associated with altered joint kinematics, reduced walking speed, and difficulties in foot clearance (Kinsella and Moran, 2008; Fock et al., 2009; Boudarham et al., 2013; Schwachmeyer et al., 2013; Manca et al., 2014). Poor foot placement control can also lead to instability during gait and an increased risk of falling (Dean and Kautz, 2015). During toe-walking, stability is reduced because of the smaller contact area between the foot and the ground (Perry et al., 2003). Furthermore, several studies have reported complaints of fatigue and pain during walking tasks in toe-walkers (Balemans et al., 2015; Alriksson-Schmidt and Hägglund, 2016; Eken et al., 2019), which may lead to a reduced health-related quality of life (HR-QoL) (Williams and Haines, 2015). A better understanding of the energetic demands associated with abnormal gait patterns could help establish intervention strategies that prevent fatigue and the deterioration of gait (Opheim et al., 2009; Lundh et al., 2018).

Toe-walkers present alterations in gait kinematics and kinetics at various joints in the lower limb (Winters et al., 1987; Kelly et al., 1997; Schlough et al., 2020). These kinematic changes are associated with premature and prolonged electromyographic (EMG) activity of the ankle plantarflexors (Kalen et al., 1986; Perry et al., 2003). Similar lower-limb joint kinetics and EMG activities were also observed in able-bodied subjects during voluntary toe-walking (Davids et al., 1999; Kerrigan et al., 2000; Perry et al., 2003; Romkes and Brunner, 2007). Romkes and Brunner (Romkes and Brunner, 2007) observed similar gastrocnemius and tibialis anterior EMG activity between voluntary and obligatory unilateral toe-walking, suggesting that the altered activations of these muscles might be required to meet the altered kinetic demands at the ankle and could therefore be partially regarded as muscle activity which is necessary in order to toe-walk. Kerrigan et al. (Kerrigan et al., 2000) observed a reduction in peak joint moments at the ankle and the knee during toe-walking, suggesting that it might require less muscle strength compared to normal a heel-to-toe gait. Perry et al. (Perry et al., 2003), on the other hand, observed an increase in mean and peak EMG activity of the plantarflexors during voluntary toe-walking, despite a reduction in peak internal plantarflexion moment – and therefore in the expected plantarflexor muscle forces. The increase in muscle EMG intensity was thought to be a consequence of a reduction in the force-generation capacity of the calf muscles when the ankle was in a plantarflexed position, in line with previous studies (Herman and Bragin, 1967; Hoy et al., 1990). Computational approaches have also been used to characterize the different contributions of the lower-limb muscles to body weight support and propulsion, showing that not only the ankle plantarflexors but also knee extensors, knee flexors, and hip extensors present an altered function during toe-walking (Sasaki et al., 2008).

Gait analysis has evolved over recent years, notably through the use of musculoskeletal modeling in clinical settings (Smith et al., 2021). Musculoskeletal modeling represents a valuable non-invasive tool to estimate the internal body loads, which could not be directly measured otherwise (Vigotsky et al., 2019; Imani Nejad et al., 2020). Modeling estimates showed good agreement with experimentally measured quantities, such as joint loads from instrumented prostheses and muscle activations from EMGs (Lund et al., 2012; Marra et al., 2014; De Pieri et al., 2018, 2019; Lunn et al., 2020; Sun et al., 2020). Through conventional gait analysis and inverse dynamics, we are able to compute kinetic parameters such as net joint moments and joint powers, which provide us insight into the function of various muscle groups in healthy and pathological gait patterns (Sloot and van der Krogt, 2018). With current musculoskeletal modeling approaches, however, it is possible to additionally predict how the load is shared across individual muscles during a given motion (Erdemir et al., 2007; Andersen, 2021). This approach is particularly useful for the analysis of human locomotion (Sylvester et al., 2021) and reveals important information about the functional role of specific muscles, particularly of the biarticular ones (Thelen et al., 2011; Seth et al., 2018). Therefore, analyzing the muscular demands associated with toe-walking using musculoskeletal modeling could provide meaningful information for the clinical management of conditions characterized by this walking pattern.

Furthermore, a synthetic parameter that could provide an insight into the required muscular demands during gait is the support moment. This parameter was first introduced by D. Winter in 1980 and was defined as the sum of the sagittal extensive moments at the ankle, knee, and hip joints (Winter 1980). The support moment quantifies how much the limb is pushing away from the ground and can be used to estimate the muscular demands necessary to prevent a collapse of the stance limb during walking (Winter 2009). In 2000, A.L. Hof provided a mechanical interpretation for this concept, demonstrating that a slightly reformulated definition of the support moment was responsible for preventing the collapse of the knee due to external forces (Hof, 2000). The support moment seems well-suited to investigate the required overall extensive muscular effort to maintain stance limb stability in pathological populations (Wyers et al., 2021).

The aim of this study was to understand and characterize, from a biomechanical perspective, gait deviations occurring during voluntary unilateral toe-walking, compared to normal gait, in a cohort of healthy volunteers. In particular, the focus was to describe the functional contributions of the major flexor and extensor muscles to the kinetics of gait. For this purpose, required muscle contributions to the joint sagittal moments were calculated using musculoskeletal modeling. The analysis of healthy individuals provides a unique insight on the muscular demands solely related to the specific gait pattern, independently of any underlying neuromuscular control disorder. As toe-walking alters the alignment of the stance limb relatively the ground reaction forces (GRF), functional adaptations for posture and movement control are expected in the lower-limb muscles to counteract these external forces. It was therefore hypothesized that toe-walking is associated with higher demands on the lower-limb muscles compared to normal walking. Additionally, the support moment is suggested as a synthetic indicator of the overall muscular demands during stance, and further clarifications about its physical meaning are provided.

## Materials and Methods

### Participants and Gait Analysis

Nine healthy adult subjects (four males, five females; age 30.1 ± 3.7 years) without a history of neurologic or orthopedic disorders underwent kinematic (motion capture system: Vicon Motion Systems Ltd., Oxford, UK) and kinetic (force platforms: Kistler Group, Winterthur, CH) 3D gait analysis, as part of a previous study (Romkes and Brunner, 2007). Gait data were acquired barefoot at a self-selected speed using the Plug-in Gait lower-body marker-set (Kadaba et al., 1990). One of the ten healthy subjects originally included in Romkes and Brunner (2007) was excluded due to an incomplete set of markers. Subjects were first tested during normal walking and were then shown a sagittal plane video of a patient with unilateral CP. They were asked to mimic the unilateral toe walking pattern seen in the video. The subjects practiced until obtaining a reproducible pattern, while care was taken by the investigators to ensure that knee and ankle positions were correct. Four to six walking trials were measured for each walking modality per subject. Further details on the experimental data collection can be found in Romkes and Brunner (2007).

### Musculoskeletal Modeling

Marker trajectories and ground reaction force (GRF) data were used as input for an inverse dynamics analysis in the AnyBody Modeling System (AnyBody Technology, Denmark) (Damsgaard et al., 2006). Individual models for each subject were created from a detailed musculoskeletal model of the lower limb (Carbone et al., 2015; De Pieri et al., 2018), which was scaled to match the overall anthropometrics and the marker data collected during a standing reference trial (Lund et al., 2015).

Each hip joint was modeled as a 3-degrees of freedom (DOF) ball-and-socket joint, while knee and talocrural joints were modeled as 1-DOF hinges. The position of the patella was defined as a function of the knee flexion angle, while the motion of the subtalar joint was restricted due to the reduced number of markers on the foot segment (one heel- and one toe-marker). Muscles were modeled as Hill-type actuators, with calibrated tendon slack-length (Heinen et al., 2016) and instantaneous muscle strength that followed force-length and force-velocity relationships (Hill, 1938; Zajac, 1989; Andersen, 2021).

Joint kinematics were first computed from the measured marker trajectories (Andersen et al., 2009) and were reported in anatomical coordinate systems according to the International Society for Biomechanics’ (ISB) recommendations (Wu et al., 2002). An inverse dynamics analysis, based on a third-order-polynomial muscle recruitment criterion, was then performed to calculate the required muscle activations and forces, as well as the resulting joint moments (Andersen, 2021). At each joint level, the internal net sagittal moment was calculated in the proximal coordinate system according to ISB recommendations (Derrick et al., 2020). In each lower-limb joint, sagittal plane rotations were unconstrained, therefore the internal sagittal net joint moment is equal to the sum of the sagittal moments generated by all the muscles spanning the joint:

$$M_{joint}^{sagittal}=\underset{m_{j}=joint-spanning\;muscles}{\sum }M_{m_{j}}^{sagittal}.$$ (1)

The contribution of each joint-spanning muscle to the joint net sagittal moment was computed as the product of the force exerted by the muscle times the distance of its instantaneous line of action from the center of rotation of the joint (De Pieri et al., 2018). For biarticular muscles, the contributions to the net sagittal moments around both joints were computed. Additionally, the force exerted by the quadriceps muscles on the patella and the force transmitted by the triceps surae to the Achilles tendon were calculated.

### Support and Progression Moments

The support moment $M_{S}$ was calculated as a weighted sum of the lower-limb joints sagittal extensive moments, according to the definition by A.L. Hof (Hof, 2000), in which the individual sagittal moments around the ankle ( $M_{A}$ ), knee ( $M_{K}$ ), and hip ( $M_{H}$ ), were considered positive when extensive. This definition of the support moment is equal to the component of the GRF acting in the direction of the hip-ankle line ( $F_{P}$ ) times knee eccentricity ( q ):

$$M_{S}=\frac{1}{2}M_{A}+M_{K}+\frac{1}{2}M_{H}=F_{P}*q.$$ (2)

As a corollary of this interpretation, it was suggested that the component of the GRF acting transversally to the direction of the hip-ankle line ( $F_{T}$ ) is determined by the difference between hip and ankle sagittal moments divided by the distance between hip and ankle ( p ). We defined the difference between hip and ankle moments as progression moment $M_{P}$ :

$$M_{P}=M_{hip}-M_{ankle}=F_{T}*p.$$ (3)

p and q were calculated as a function of the knee flexion angle using trigonometric relationships, under the assumption that thigh and shank have equal lengths. The complete geometrical derivation of these relationships is reported in Hof (2000).

**FIGURE 1:**
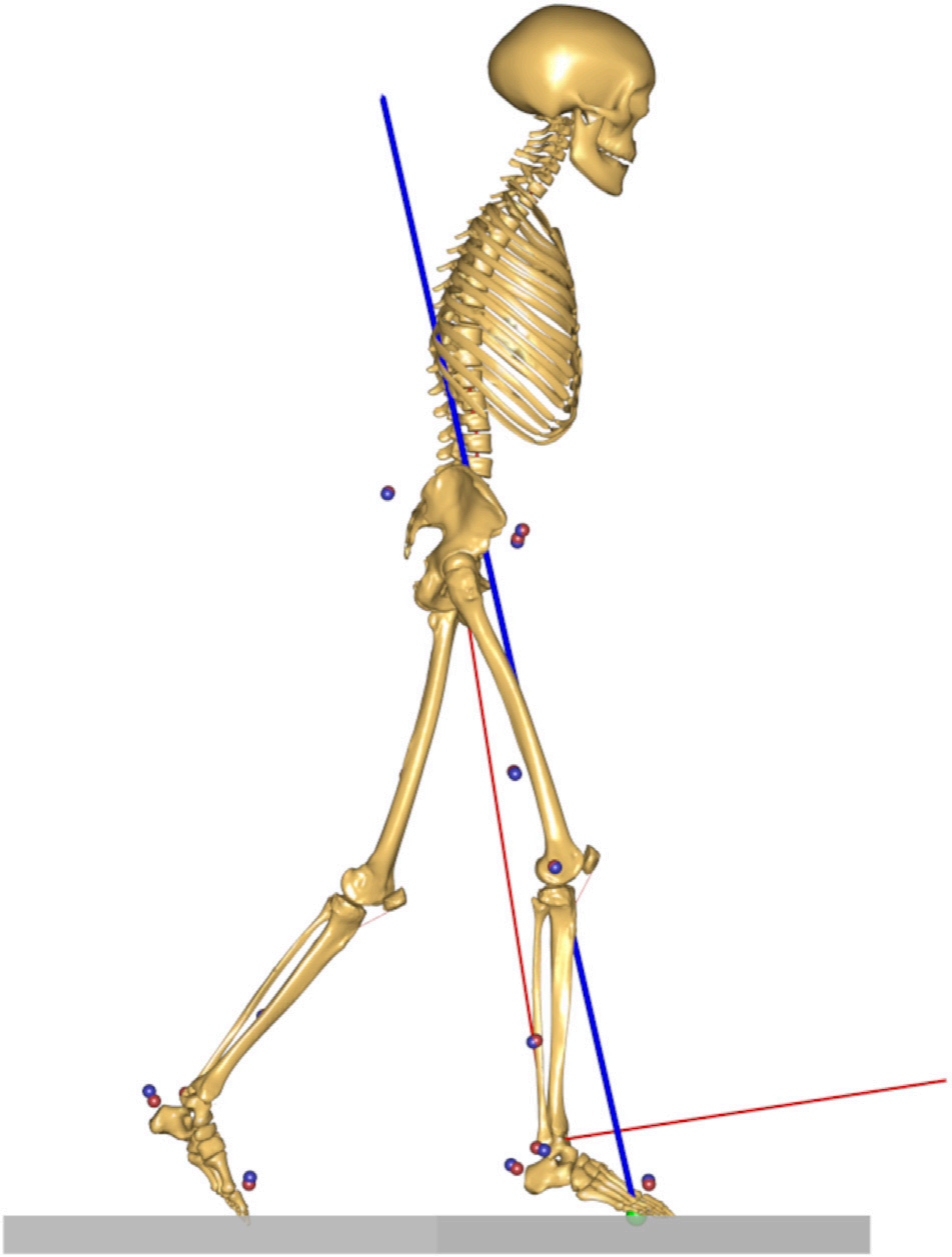
Ground reaction force vector (in blue) and the newly defined reference frame (in red) with the vertical axis connecting the ankle and the hip joint centers, and the anterior-posterior axis transversal to the hip-ankle line.

$F_{p}$ and $F_{t}$ were calculated by projecting the GRF vector into a newly defined reference frame, in which the vertical axis passes through the ankle and the hip joint centers. The mediolateral axis was parallel to the floor and perpendicular to the gait direction (defined by the floor-projection of the heel marker in two consecutive ipsilateral foot strikes), and the anteroposterior axis was defined as the cross-product of vertical and mediolateral ones (Figure 1).

The support and progression moments $M_{S}$ and $M_{P}$ , calculated as the sum or difference of the individual joint moments as described previously, were then compared to the physical quantities $F_{P}*q$ and $F_{T}*p$ , respectively. Additionally, muscle contributions to the support and the progression moments were calculated by combining (Eq. 1) into (Eqs 2, 3), respectively:

$$M_{S}=\;\frac{1}{2}\sum_{m_{A}}M_{m_{A}}+\sum_{m_{K}}M_{m_{K}}+\;\frac{1}{2}\sum_{m_{H}}M_{m_{H}}=\;\left(\frac{1}{2}M_{Sol_{A}}+0+0\right)+\left(\frac{1}{2}M_{Gas_{A}}+M_{Gas_{K}}+\;0\right)+\;\left[\ldots \right],$$ (4)

$$M_{P}=\;\sum_{m_{H}}M_{m_{H}}-\sum_{m_{A}}M_{m_{A}}=-M_{Sol_{A}}-M_{Gas_{A}}-M_{TibAnt_{A}}+M_{Glut{\mathrm{Max}}_{H}}+M_{Hams_{H}}+\;\left[\ldots \right].$$ (5)

### Data Analysis

Gait trials were processed and analyzed through the toolkit AnyPyTools (Lund et al., 2019) in the *Python* programming language (*Python* Software Foundation). The analysis of joint kinematics and joint kinetics, including muscle contributions, was focused on the sagittal plane as the plane of forward motion. Joint moments, muscle contributions to the joint moments, and support and progression moments were normalized by body mass. The muscle forces acting on the patella and the Achilles tendon were normalized by body weight (BW). Kinematic data (angles) were time-normalized during the gait cycle (GC) from foot strike (0%) to foot strike (100%), while kinetic data (moments and forces) were time-normalized for the duration of the loaded stance phase (ST), from foot strike (0%) to foot off (100%). Averages per subject were then calculated based on the four to six collected trials.

### Statistical Parametric Mapping Analysis

Differences between normal walking and voluntary toe-walking were analyzed through statistical parametric mapping (SPM; www.spm1D.org, v0.43) (Friston et al., 1994; Pataky, 2012) for joint kinematics, joint moments, muscle contributions to the joint moments, muscle forces, and for support and progression moments. Due to the reduced number of tested subjects (*n* = 9), non-parametric (Pataky et al., 2015) two-tailed paired *t*-tests were used to identify statistically significant differences between the two walking modalities.

The comparison between support and progression moments and their respective physical equivalents, $F_{P}*q$ and $F_{P}*p$ , was carried out through non-parametric, two-tailed, two-sample t-tests.

The output test statistic—SnPM{t}—was evaluated at each point of GC or ST. The significance level was set at *α* = 0.05, and the corresponding critical thresholds—t*—were calculated based on the temporal smoothness of the input data through random field theory. The probability that similar suprathreshold regions would have occurred from equally smooth random waveforms was then calculated. In the interest of clarity, only differences that were statistically significant for more than 2% of GC or ST are discussed.

## Results

### Lower-Limb Kinematics

**FIGURE 2:**
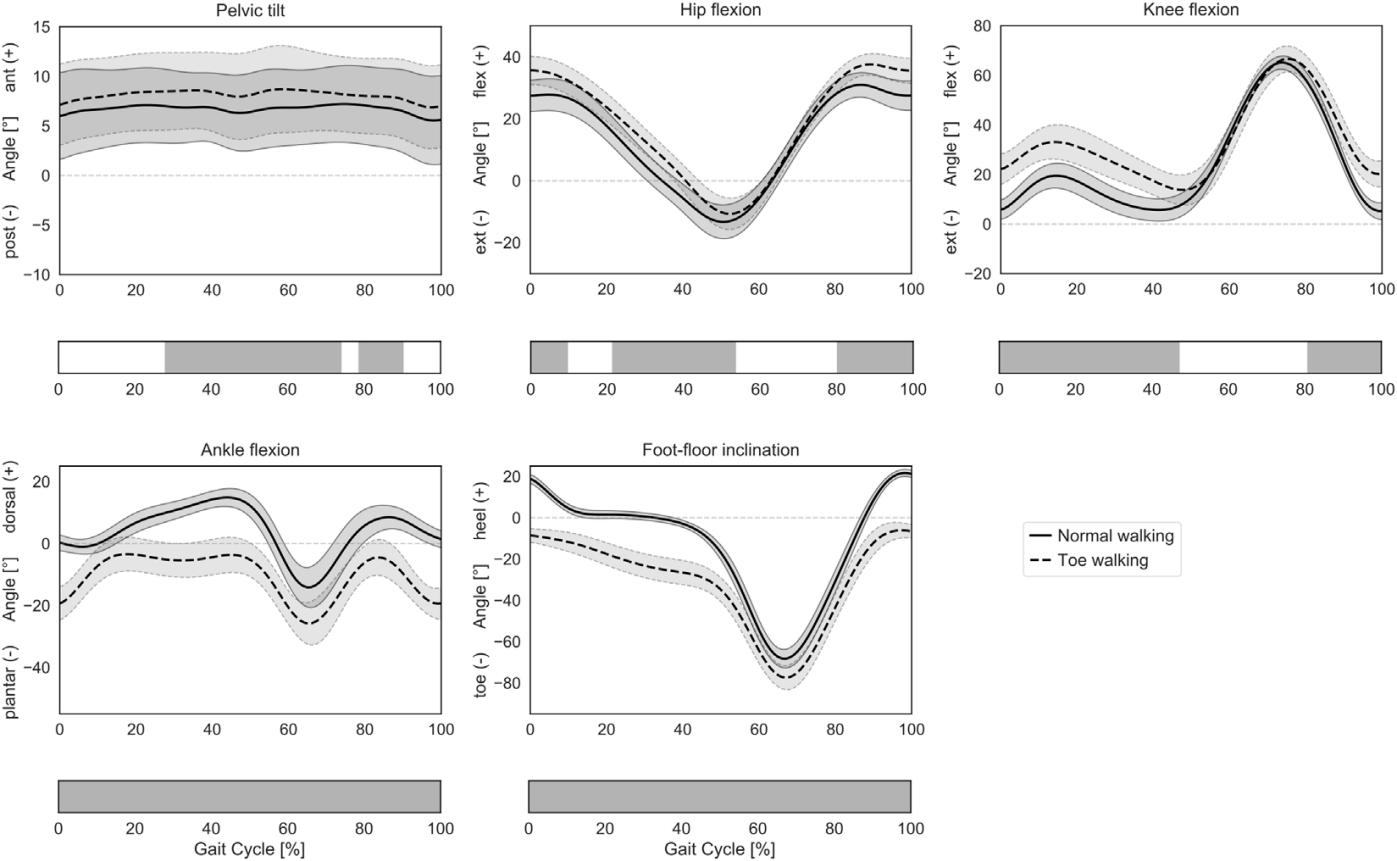
Lower-limb sagittal plane kinematics during GC. Mean ± 1SD pelvic tilt, hip flexion, knee flexion, ankle flexion, and foot-floor inclination angles are reported as solid lines during normal walking and as dashed lines during voluntary toe-walking. Phases of GC for which a statistically significant difference in the SPM paired t-tests was found are indicated as grey bars below each subplot.

The subjects participating in this study were able to successfully reproduce a toe-walking pattern similar to the one they were shown in a video of a unilateral CP patient. In the sagittal plane, their voluntary toe-walking pattern was characterized by an initial toe-contact, and the foot-floor inclination angle significantly differed from their normal gait pattern throughout the GC, with the heel never touching the ground (Figure 2). During toe-walking, the ankle was significantly more plantarflexed during the whole GC, the knee was more flexed from mid-swing until terminal stance (81–47% GC), the hip was more flexed from mid-swing until loading response and during mid-stance (80–10% and 21–54% GC), and the pelvis was more anteriorly tilted from mid-stance until mid-swing (28–74% and 79–90% GC).

### Joint Moments and Muscle Contributions

When walking on their toes, the subjects also presented statistically significant differences in the internal net sagittal joint moments compared to their normal gait pattern. These kinetic differences translated into altered required moment contributions in the sagittal plane from the muscles spanning each joint.

**FIGURE 3:**
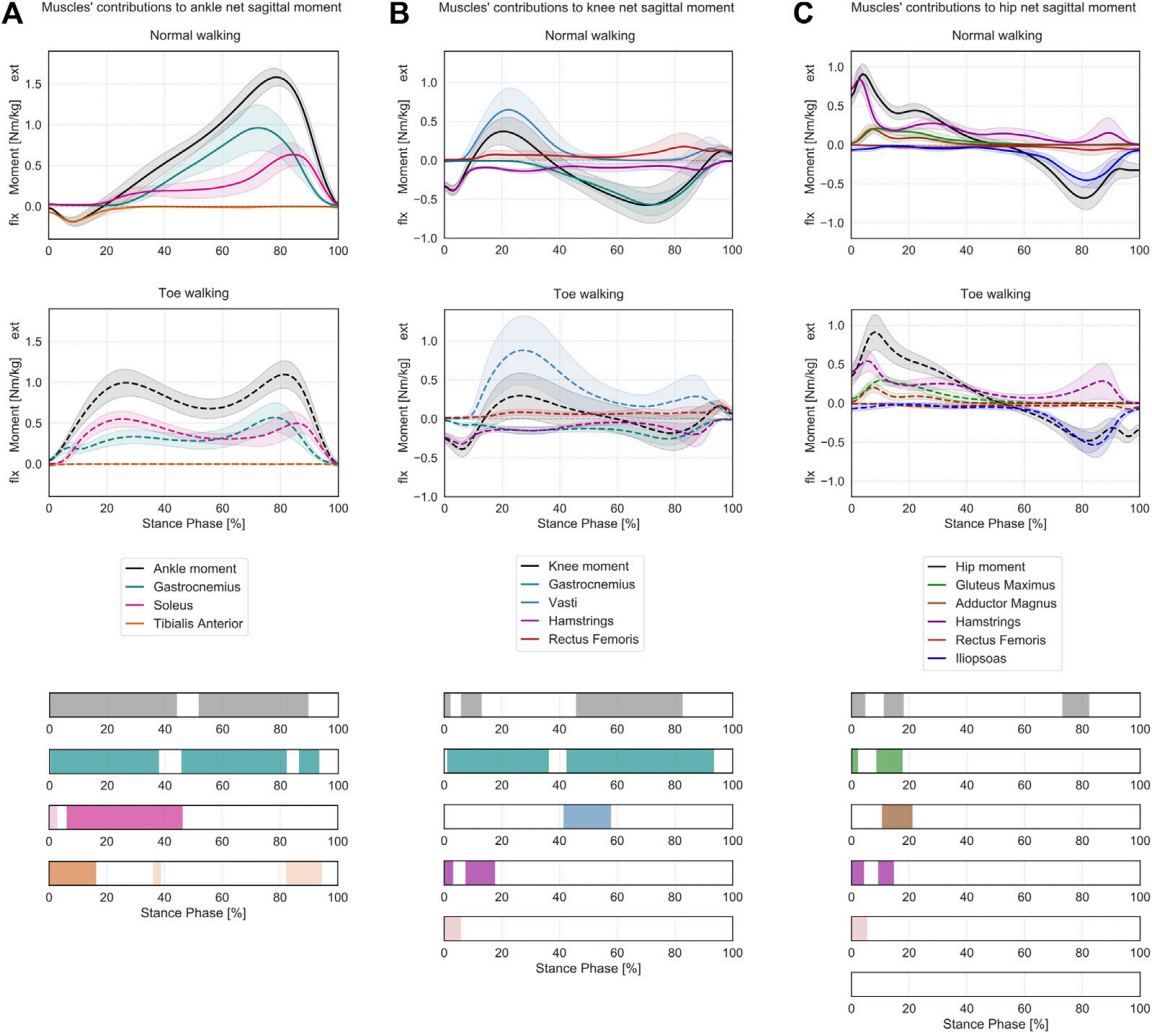
Lower-limb sagittal plane kinetics during ST. Mean ± 1SD ankle **(A)**, knee **(B)** and hip **(C)** sagittal net internal moments (black/grey) and the sagittal moment contributions of the relative joint-spanning muscles (colored) are reported as solid lines during normal walking and as dashed lines during voluntary toe-walking. Phases of ST for which a statistically significant difference in the SPM paired *t*-tests was found are indicated as colored bars at the bottom. ST phases in which statistically significant differences were observed but the moment contributions were characterized by negligible magnitudes are reported in lighter colors.

At the ankle (Figure 3A), toe-walking was associated with a significantly larger net extension (plantarflexion) moment during loading response and mid-stance (0–44% ST) and with a reduced extension moment during terminal-stance and push-off (52–90% ST). As a consequence of the altered kinetics, toe-walking required an earlier and larger extensive contribution during loading response and mid-stance from the gastrocnemius (0–38% ST) and the soleus (6–46% ST), while the gastrocnemius also provided a reduced contribution to the peak extension moment during terminal-stance and push-off (46–82% and 86–93% ST). The tibialis anterior did not provide any flexion (dorsiflexion) contribution during initial contact and loading response (0–16% ST) when toe-walking. Additional statistically significant differences in soleus (0–3% ST) and tibialis anterior (36–38% and 82–94% ST) were characterized by negligible magnitudes in both walking modalities.

At the knee (Figure 3B), the subjects presented a longer-lasting net flexion moment during loading response, decreased during 0–2% ST and increased during 6–13% ST when toe-walking. From mid- to terminal-stance (46–83% ST), the flexion moment was significantly reduced. No statistically significant differences were found during early mid-stance when the net sagittal moment was extensive for normal walking and toe-walking. In terms of muscles’ contributions to the knee net sagittal moment, the gastrocnemius provided a larger flexion moment during the first half from loading response (1–36% ST) and a reduced flexion moment during mid-stance to push-off (42–93% ST) when toe-walking. A significantly larger extension moment was required from the vasti during mid-stance (41–58% ST) when walking on the toes, while their extensive contribution was almost null during this phase while normal walking. The peak extension moment from the vasti occurred for both walking modalities during loading response and was characterized by a larger mean value and a broader variability during toe-walking, albeit not significantly different (0.88 ± 0.44 Nm/kg at 23% ST during toe walking vs 0.65 ± 0.28 Nm/kg at 22% ST during normal walking). The hamstrings provided a reduced but longer-lasting flexion contribution during loading response (lower during 0–3% ST and increased during 8–18% ST). The rectus femoris provided a significantly larger extension contribution during initial contact (0–6% ST) but of negligible magnitude.

At the hip (Figure 3C), toe-walking was characterized by a larger and delayed net extension moment during loading response (lower during 0–5% ST and increased during 11–18% ST) and by a reduced net flexion moment during terminal-stance (73–82% ST). When toe-walking, the gluteus maximus provided a larger contribution to the extension moment during initial stance (0–2% and 8–17% ST), similarly to the adductor magnus (11–21% ST), while the hamstrings provided a reduced extensive contribution during initial contact (0–4% ST) and a slightly increased one during loading response (11–21% ST). The significant flexing contribution of the rectus femoris (0–5% ST) was of negligible magnitude.

### Support Moment

**FIGURE 4:**
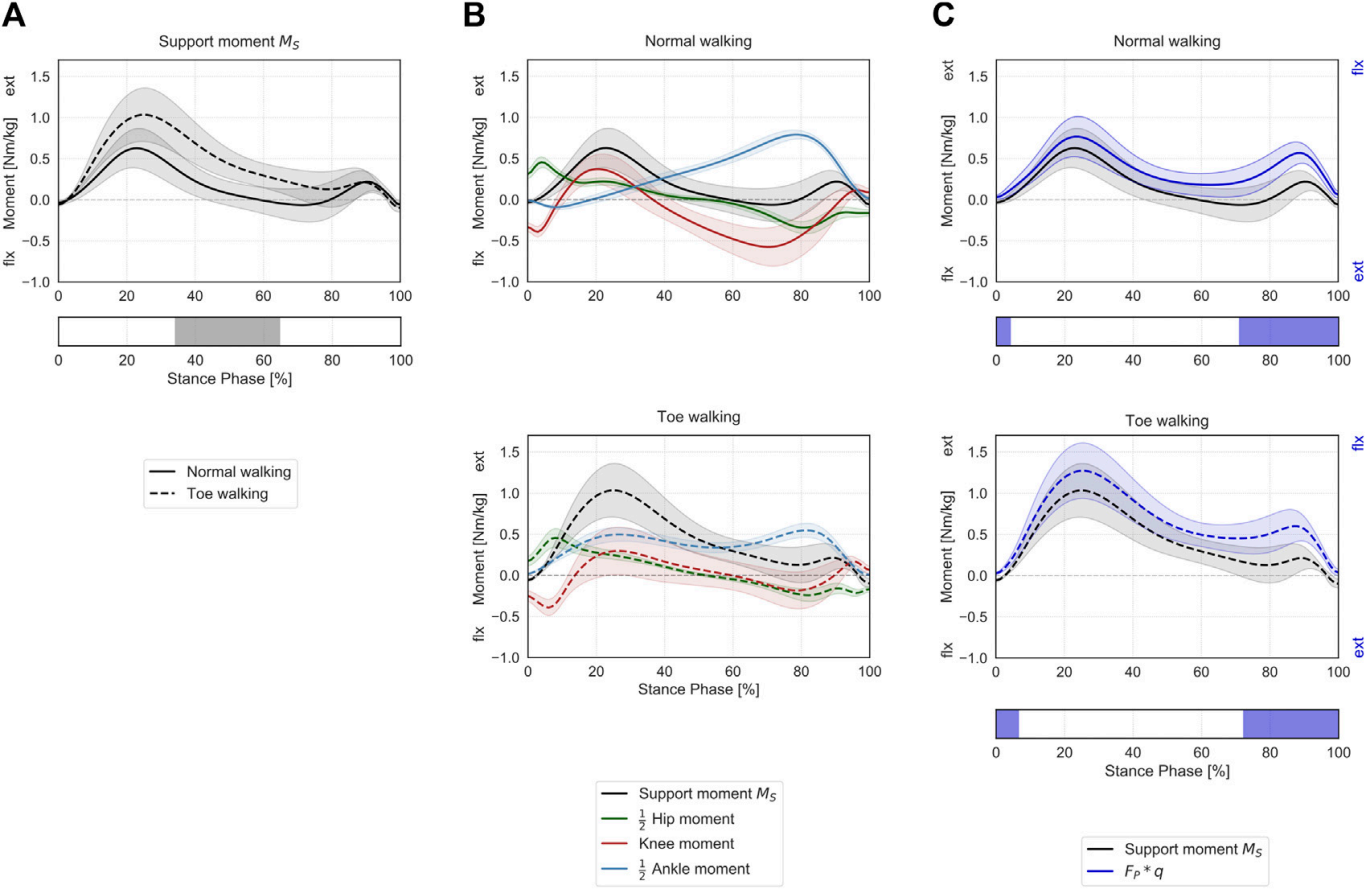
Support moment in the sagittal plane during ST. **(A)** Mean ± 1SD support moment (black/grey) is reported during normal walking (solid line) and toe-walking (dashed line). The phase of ST for which a statistically significant difference in the SPM paired *t*-test was found is indicated as a grey bar below. **(B)** Mean ± 1SD support moment (black/grey), ankle (blue), knee (red), and hip (green) internal net sagittal moments, weighted according to the definition by A.L. Hof (Hof, 2000), are reported for normal walking (top, solid lines) and toe-walking (bottom, dashed lines). **(C)** Comparison between support moment and the external forces acting on the stance limb. Mean ± 1SD support moment (black/grey) compared to the product of the GRF component acting in the direction of the hip-ankle line FP times knee eccentricity q (violet-blue), during normal walking (solid line, top) and toe-walking (dashed line, bottom). The support moment is acting in the opposite direction compared to the moment produced by the GRF. The phases of ST for which a statistically significant difference in the SPM two-sample *t*-test was found are indicated as violet-blue bars below.

Toe-walking was associated with a significantly higher support moment during mid-stance (34–65% ST) (Figure 4A), as a consequence of the altered joint sagittal moments (Figure 4B).

The support moment calculated according to Hof’s formulation showed a similar trend and comparable magnitude with the product of the GRF component acting in the direction of the hip-ankle line ( $F_{P}$ ) times knee eccentricity ( q ) (Figure 4C). Statistically significant differences between $M_{S}$ and $F_{P}*q$ were found during the initial transition phase and pre-swing (0–4% and 71–100% ST for normal walking, 0–6% and 72–100% ST for toe-walking). This confirms that a weighted sum of the three lower-limb joint moments is responsible for preventing the collapse of the knee during stance.

When looking at the contribution of the major lower-limb muscles to the support moment during normal walking (Figure 6A), it emerges that the hamstrings briefly provide extensive vertical support during initial heel-strike while the other major hip extensors – gluteus maximus and adductor magnus – immediately follow and provide some support throughout the initial phases of stance. The largest contributor during loading response is the vasti, with a synergistic contribution from the rectus femoris. Approximatively around 20% ST, the soleus also starts providing a positive contribution to the support moment, and it becomes the dominant contributor from mid-stance until push-off. From mid-to terminal-stance, the rectus femoris also plays a significant supporting role. During terminal stance, the mean support moment across the cohort becomes negative (∼60–80% ST), corresponding with the peak knee flexion moment (Figure 4B). During this phase, the hamstrings and the gastrocnemius provide a net negative contribution to the support moment. During pre-swing, the vasti work again synergistically with the rectus femoris and the soleus to provide a net positive support moment.

During toe-walking, the hamstrings provide a prolonged negative contribution during the initial foot contact until loading response. This is counteracted by a synchronous positive contribution from the gluteus maximus and the adductor magnus. The soleus is required to provide a positive and large contribution already during loading response and lasting until push-off. The vasti remain the largest contributors to the support moment, and their activation is also continuously required from load acceptance throughout ST. The rectus femoris contribution is also qualitatively reduced, particularly during push-off. The mean support moment remains positive throughout ST when toe-walking, despite a negative contribution from the hamstring during terminal stance. The gastrocnemius provides a small positive contribution during terminal stance.

### Progression Moment

**FIGURE 5:**
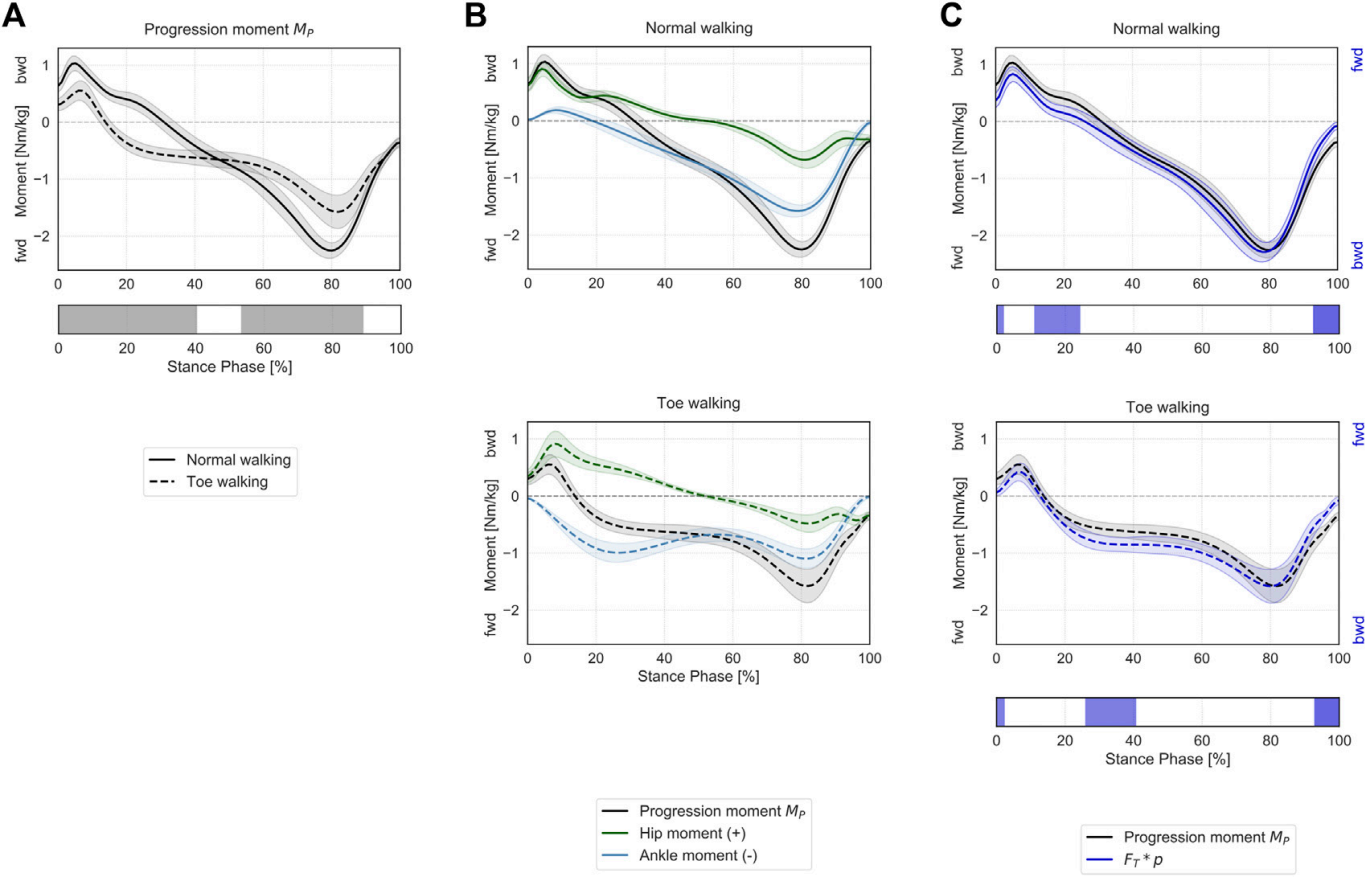
Progression moment in the sagittal plane during ST. **(A)** Mean ± 1SD progression moment (black/grey) is reported during normal walking (solid line) and toe-walking (dashed line). The phases of ST for which a statistically significant difference in the SPM paired *t*-test was found are indicated as grey bars below. **(B)** Mean ± 1SD progression moment (black/grey), ankle (blue), and hip (green) internal net sagittal moments, weighted according to the definition derived from A.L. Hof (Hof, 2000), are reported for normal walking (top, solid lines) and toe-walking (bottom, dashed lines). **(C)** Comparison between support moment and the external forces acting on the stance limb. Mean ± 1SD progression moment (black/grey) compared to the product of the GRF component acting transversally to the direction of the hip-ankle line FT times the distance between hip and ankle p (violet-blue), during normal walking (solid line, top) and toe-walking (dashed line, bottom). The progression moment is acting in the opposite direction compared to the moment produced by the GRF. The phases of ST for which a statistically significant difference in the SPM two-sample *t*-test was found are indicated as violet-blue bars below.

Toe-walking was associated with a significantly reduced peak backward progression moment during the initial foot contact and loading response (0–40% ST) (Figure 5A). The mean progression moment becomes forward-oriented at 14% ST during toe-walking, compared to normal walking when this transition occurs at 31% ST. This was determined by an earlier forward contribution by the ankle sagittal moment (Figure 5B). From mid- to terminal-stance, toe-walking was characterized by a significantly reduced peak forward progression moment (53–89% ST).

The progression moment, defined as a corollary of Hof’s interpretation, showed a similar trend and comparable magnitude with the product of the GRF component acting transversally to the direction of the hip-ankle line ( $F_{T}$ ) times the distance between hip and ankle ( p ) (Figure 5C). Statistically significant differences between $M_{P}$ and $F_{T}*p$ were found during the transitions between stance and swing phases (0–2% and 92–100% ST for normal walking, 0–2% and 93–100% ST for toe-walking), as well as during 11–24% ST for normal walking and 26–41% ST for toe-walking. This suggests that only ankle- and hip-spanning muscles are counteracting the external forces acting transversally to the stance leg.

**FIGURE 6:**
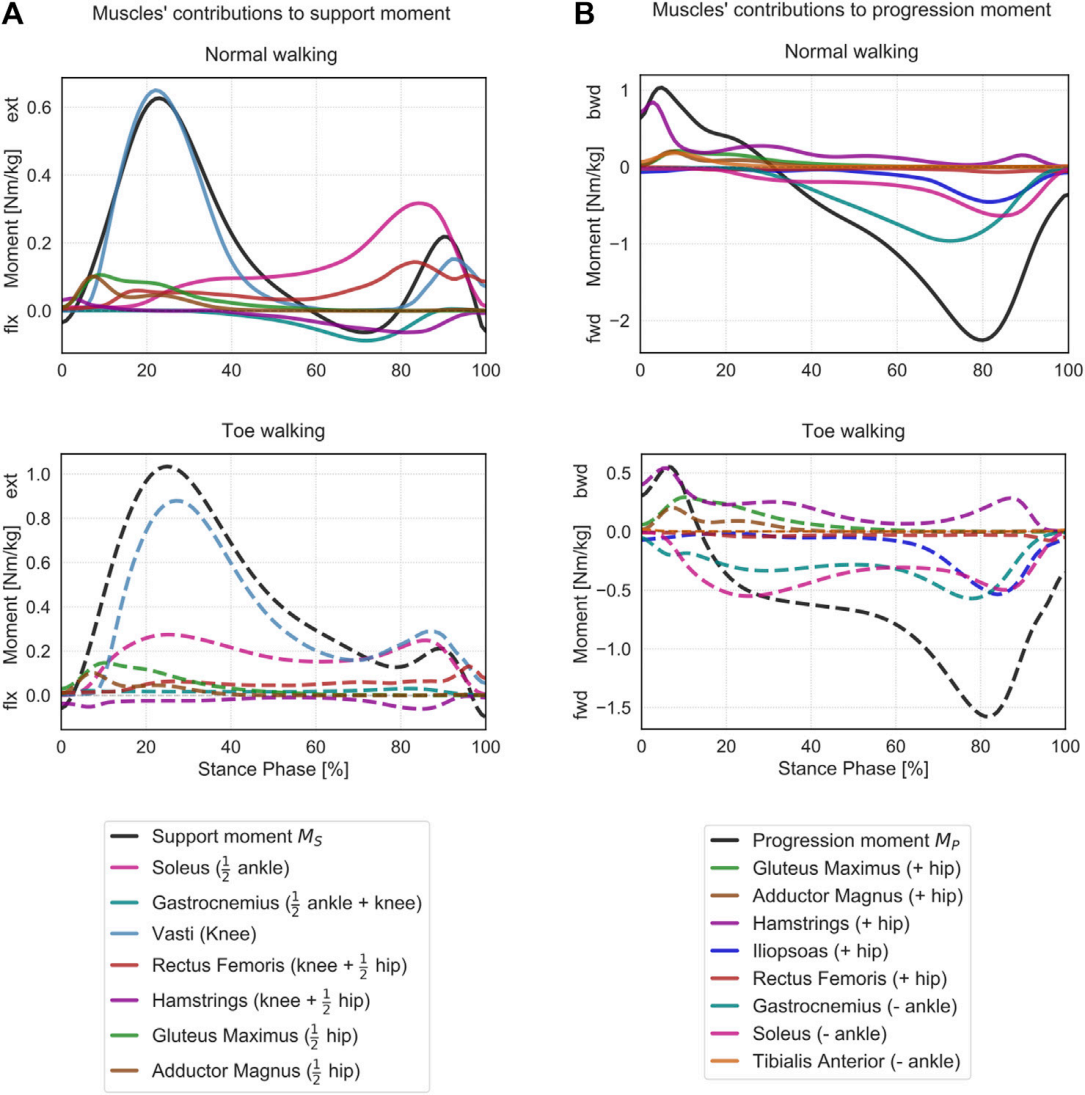
Contributions of the lower limb muscles to support and progression moments. **(A)** Mean support moment (black) and sagittal moment contributions of the major lower-limb muscles are reported for normal walking (top, solid lines) and toe-walking (bottom, dashed lines). For each muscle, the contribution to the support moment is calculated as a weighted sum of its contributions to the joints it spans, as indicated in Eq. 4. Weighing factors are reported in brackets ( +1/2 for ankle and hip,  +1 for the knee). **(B)** Mean progression moment (black) and sagittal moment contributions of the major lower-limb muscles are reported for normal walking (top, solid lines) and toe-walking (bottom, dashed lines). For each muscle, the contribution to the progression moment is calculated as a weighted contribution to the joint it spans, as indicated in Eq. 5. Weighing factors are reported in brackets ( −1 for the ankle, +1 for the hip).

The muscles’ contributions to the progression moment were qualitatively different between normal walking and toe-walking (Figure 6B). During normal walking, in order to counteract the forward-oriented $F_{T}*p$ (Figure 5C), the hamstrings provide a backward contribution during the initial heel-strike. Their contribution remains present albeit reduced during most of the stance phase. The other hip extensors – gluteus maximus and adductor magnus – immediately follow and provide a backward contribution during loading response, together with the tibialis anterior. This highlights a synergistic effect of hip extensors and ankle dorsiflexors in early stance in slowing down the anterior motion of the leg. From mid-stance, the ankle plantarflexors – soleus and gastrocnemius – provide a forward contribution to the progression moment (counteracting the posteriorly oriented $F_{T}*p$ ), as does the iliopsoas during pre-swing. The forward contribution of the rectus is smaller compared to the iliopsoas. There is an overall synergistic action of ankle plantarflexors and hip flexors in rotating the leg forward.

During toe-walking, the hamstring provided a relatively larger backward contribution for a prolonged period of ST. The lack of tibialis anterior backward contribution is compensated by a larger relative contribution of all hip extensors during early stance. At the same time, the soleus and the gastrocnemius provide a forward contribution already during the early stance. The hip extensors and the ankle plantarflexors are therefore acting antagonistically during the initial phases of ST when toe-walking. During mid-stance to late stance, the forward contribution to the progression moment of the plantarflexors remains substantial, and it is strengthened by a synergistic effect of the iliopsoas and to a smaller extent of the rectus femoris during pre-swing.

### Muscle Forces on Patella and Achilles Tendon

**FIGURE 7:**
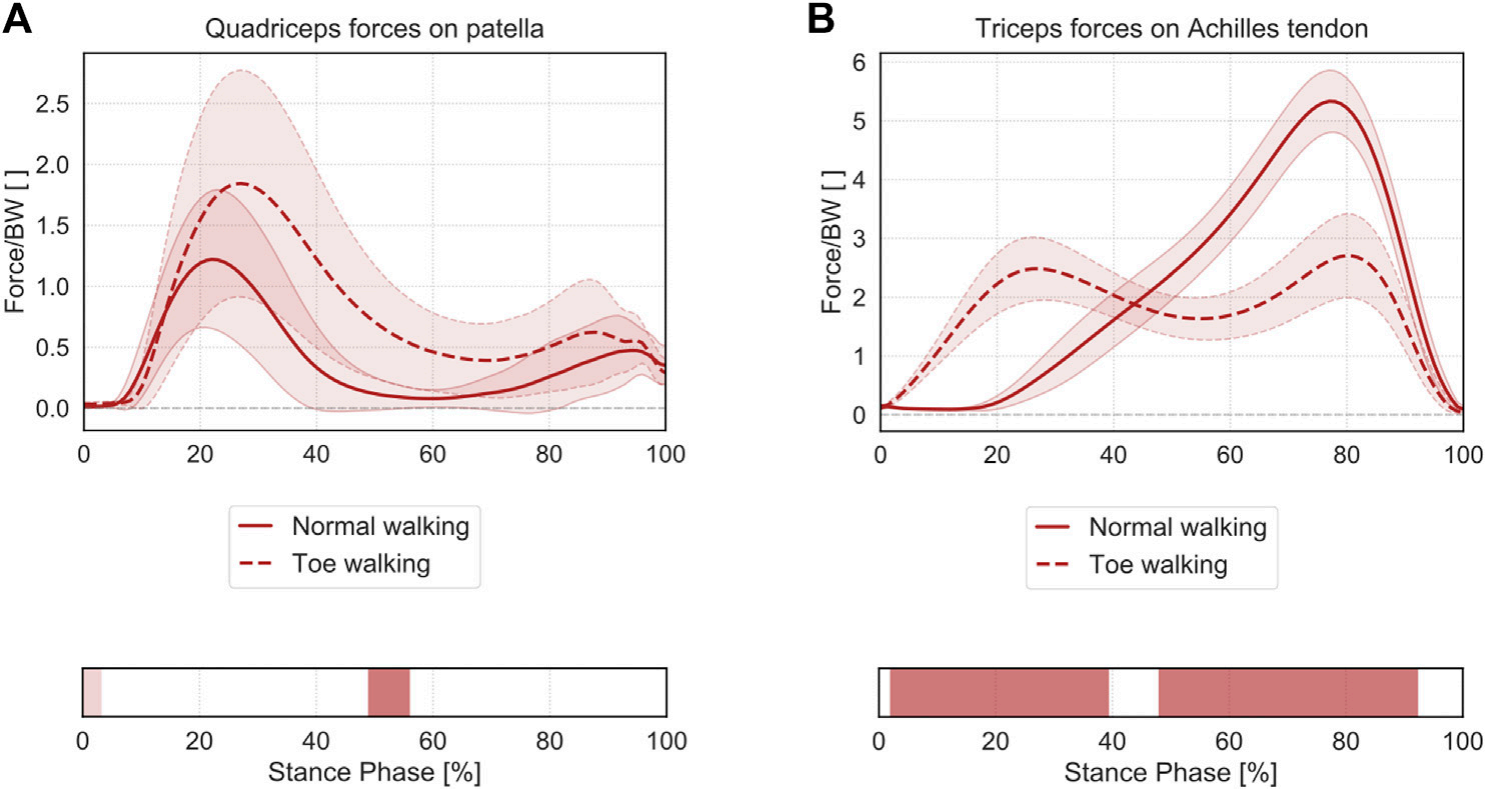
Mean ± 1SD magnitude of the muscle forces transmitted from the quadriceps muscle to the patella **(A)** and from the triceps surae to the calcaneus through the Achilles tendon **(B)**, during normal walking (solid lines) and toe-walking (dashed lines), normalized to body weight (BW). The phases of ST for which statistically significant differences in the SPM paired *t*-tests were found are indicated as red bars below each subplot. ST phases in which statistically significant differences were observed but the muscle forces were characterized by negligible magnitudes are reported in a lighter tint.

Toe-walking was associated with significantly larger muscle forces exerted by the quadriceps to the patella during mid-stance (49–56% ST) (Figure 7A). The significantly higher muscle forces during initial foot contact (0–3% ST) were of negligible magnitude.

Regarding the muscle forces exerted from the triceps surae to the calcaneus (Figure 7B), toe-walking was also characterized by a prolonged force transmission through the Achilles tendon, with higher forces during early stance (2–39% ST) and a reduced peak force during terminal-stance (48–92% ST).

## Discussion

The objective of this study was to describe the gait deviations occurring in a cohort of healthy subjects when they are voluntarily walking on their toes compared to their normal heel-to-toe gait pattern. The focus was to characterize the functional adaptations of the major lower-limb muscles which are required in order to toe walk. The role and importance of each muscle to the kinetics of gait was assessed by estimating its required contribution to the joint net sagittal moments using musculoskeletal modeling. Additionally, the concept of the support moment was used to evaluate the overall muscular effort necessary to maintain stance limb stability and prevent the collapse of the knee due to external forces (winter 1980, 2009; Hof, 2000).

Compared to a normal heel-to-toe gait pattern, toe-walking was characterized by significantly different lower-limb joint kinematics and kinetics (Table 1). The altered kinetic demands at each joint translated into different required moment contributions from most flexor and extensor muscles. In particular, an earlier and prolonged ankle plantarflexion contribution was required from the soleus and gastrocnemius during most of the stance phase. On the other hand, the tibialis anterior did not provide any dorsiflexion contribution during the initial stance phase when toe-walking. At the knee, a reduced net flexion moment during mid-to terminal-stance corresponded with a significantly larger knee extension contribution required from the vasti during mid-stance, while the hamstrings provided a reduced but longer-lasting knee flexion contribution during loading response when toe-walking. All hip extensors provided a larger extensive moment during loading response. Overall, different muscular activations are therefore functionally required at every joint level in order to toe-walk compared to normal walking.

**TABLE 1:** Overview of most important differences in sagittal kinematics and kinetics occurring in toe-walking compared to normal walking during different periods of the stance phase.

Major sagittal kinematic and kinetic differences of toe-walking compared to normal walking during stance phase					
	*Initial contact*	*Loading response*	*Mid-stance*	*Terminal stance*	*Pre-swing*
*Kinematics*	Toe-contact (vs heel); Plantarflexed ankle (vs neutral); Larger knee flexion; Larger hip flexion	Toe-contact (vs heel); Plantarflexed ankle (vs dorsiflexed); Larger knee flexion; Larger hip flexion	Toe-contact (vs flat foot); Plantarflexed ankle (vs dorsiflexed); Larger knee flexion; Larger hip flexion	Toe-contact (vs flat foot); Plantarflexed ankle (vs dorsiflexed); Larger knee flexion; Larger hip flexion; Larger anterior pelvic tilt	Larger foot-floor inclination angle; Larger ankle plantarflexion; Lower peak hip extension; Larger anterior pelvic tilt
*Ankle kinetics*	Net plantarflexion (vs dorsiflexion) moment; Gastrocnemius plantarflexion contribution; Lack of tibialis anterior dorsiflexion contribution	Net plantarflexion (vs dorsiflexion) moment; Gastrocnemius plantarflexion contribution; Soleus plantarflexion contribution; Lack of tibialis anterior dorsiflexion contribution	Larger net plantarflexion moment; Prolonged and initially larger gastrocnemius plantarflexion contribution; Prolonged and larger soleus plantarflexion contribution	Lower net plantarflexion moment; Lower gastrocnemius plantarflexion contribution	Lower peak net plantarflexion moment; Lower gastrocnemius plantarflexion contribution
*Knee kinetics*	Lower net flexion moment; Lower hamstring flexion contribution	Prolonged and larger net flexion moment; Gastrocnemius flexion contribution; prolonged hamstring flexion contribution	Prolonged net extension moment; Lower gastrocnemius flexion contribution; Larger vasti extension contribution	Lower net flexion moment; Lower gastrocnemius flexion contribution	Lower gastrocnemius flexion contribution
*Hip kinetics*	Lower net extension moment; Lower hamstring extension contribution; Larger gluteus maximus extension contribution	Prolonged net extension moment; prolonged and larger hamstring extension contribution; Larger gluteus maximus extension contribution; Larger adductor magnus extension contribution		Lower net peak flexion moment	
*Support moment*			Larger extensive support moment	Larger extensive support moment	
*Progression moment*	Lower backward progression moment	Early transition backward to forward progression moment	Larger forward progression moment	Lower forward progression moment	Lower peak forward progression moment

The higher support moment during the stance phase indicates that an overall higher muscular effort is required during toe-walking to support the stance limb. The computed muscle contributions to the support moment reveal that different muscles effectively work to maintain a stable leg during different phases of walking. In particular, when toe-walking, a higher extensive contribution is required by the soleus during initial stance, and by the vasti, which present a prolonged extensive activity during mid-stance while the contribution of the rectus during pre-swing is reduced compared to normal walking. The hamstrings, which provide an extensive contribution to the support moment in a normal heel-to-toe gait pattern, are instead providing a flexing contribution during initial contact when toe-walking. These changes indicate an overall different support strategy to maintain a stable leg during the stance phase and prevent the knee from collapsing.

The analysis of the progression moment, which was derived as a corollary of A.L. Hof formulations (Hof, 2000), showed that during normal walking, forward-oriented external forces are counteracted by hip extensors and ankle dorsiflexors (initial ST) while backward-oriented external forces are balanced by activation of ankle plantarflexors and hip flexors (mid- and terminal ST). During toe-walking, this activation pattern is altered, with the hip extensors and ankle plantarflexors being simultaneously activated during the initial phases of ST. The altered activation of gastrocnemius and soleus during early stance seems to be required to maintain a force balance transversally to the leg axis when toe-walking.

Furthermore, toe-walking was characterized by significantly larger muscle forces exerted by the quadriceps to the patella and prolonged force transmission through the Achilles tendon during the stance phase.

Prolonged muscular effort and overuse may lead to fatigue, pain, and a reduced HR-QoL (Balemans et al., 2015; Williams and Haines, 2015; Alriksson-Schmidt and Hägglund, 2016; Eken et al., 2019). In particular, the higher loads required from the vasti may explain the high prevalence of knee pain and patellofemoral symptoms observed in CP patients, especially with increasing age (Jahnsen et al., 2004; Rethlefsen et al., 2015). Overactive quadriceps muscles may lead to an overstretched patellar tendon and a further loss of functionality (Villani et al., 1988; Jahnsen et al., 2004). Similarly, the prolonged force transmission through the Achilles tendon in a plantarflexed position may be associated with tendon shortening and contractures (Tardieu et al., 1989; Solan et al., 2010). Optimal treatment options should therefore aim at addressing the primary neuromuscular disorders which are associated with an altered gait pattern in order to avoid secondary long-term adverse consequences.

The restoration of a heel-to-toe gait pattern may improve the functionality and the gait efficiency of toe-walking patients while reducing the long-term risks associated with compensatory mechanisms (Brunner et al., 2021). Several treatments aiming at restoring a heel-strike pattern are available (Graham et al., 2016), ranging from conservative options, such as physical therapy (Palmer et al., 2010), casting, and orthotic devices (Totah et al., 2019), to the use local medication (Botulinum toxin-A) (Strobl et al., 2015) and bone and soft tissue corrective surgeries (McGinley et al., 2012). Physiotherapy could also benefit from a better understanding of the compensatory mechanisms to define targeted strengthening and rehabilitation programs (Damiano and Abel, 1998; Booth et al., 2018). Therefore, the choice of treatment depends on a correct understanding of the underlying pathology and on the individual biomechanics of walking.

This study was limited to a cohort of nine healthy volunteers who were asked to mimic a pathological toe-walking pattern. Through the analysis of healthy individuals, this study isolated the influence of a specific motion pattern on the optimally-required muscular loads, without additional assumptions of altered neuromuscular control (Steele et al., 2015). Musculoskeletal modeling addresses the muscle redundancy problem by identifying an optimal set of muscle activations that can provide the necessary forces and torques to reproduce an experimentally measured motion (Crowninshield and Brand, 1981; Rasmussen et al., 2001; Andersen, 2021). The timing of the predicted muscle contributions for both walking modalities in this study is in qualitative agreement with the EMG data reported for the same healthy cohort (Romkes and Brunner, 2007), further confirming the validity of the modeling predictions in a healthy cohort. These assumptions, however, might not always hold true when analyzing the motion of patients with aberrant motor control, such as those affected by CP (Veerkamp et al., 2019).

Romkes and Brunner (2007) observed similar gastrocnemius and tibialis anterior EMG activity between voluntary (healthy) and obligatory (pathological) unilateral toe-walking. These measured activations of gastrocnemius and soleus during toe-walking may therefore be interpreted as dictated by the biomechanics of toe-walking, rather than by the pathology alone. Such muscle activations, which have a direct correspondence with the measured kinematic pattern, can also be predicted through musculoskeletal modeling. It is also expected that similar gait patterns would lead to similar predicted activation patterns in healthy and pathological subjects. These predicted activations in pathological subjects could be interpreted as optimal and minimally required activity in order to perform a given motion. In reality, patients may also present additional muscle activity in the form of antagonist or stabilizing co-activations (Solomonow et al., 1988; Unnithan et al., 1996; Ikeda et al., 1998), which cannot be predicted adequately within the current musculoskeletal modeling framework (Mortensen et al., 2018). For instance, different activations of the rectus femoris during the swing phase were previously observed between voluntary and obligatory toe-walking (Romkes and Brunner, 2007). However, the kinetic analysis in this study was limited to the stance phase. A better understanding of the functional role of different muscles during swing phase motion is also necessary (Arnold et al., 2007; Brunner and Rutz, 2013).

Overall, the analysis of healthy individuals provides a unique insight into the gait-pattern-related muscular demands. Nevertheless, the generalization of these findings to a pathological population should be carried out cautiously. Despite being able to mimic a typical unilateral toe-walking pattern, the gait of the healthy participants in this study was characterized by slight differences in spatiotemporal parameters, leg length discrepancy, and joint kinematics compared to obligatory toe-walking CP patients (Romkes and Brunner, 2007). Future research should aim at investigating gait data of actual toe-walking patients characterized by different neuromuscular conditions to confirm whether they show similar kinetic patterns and required muscular demands. Other commonly-observed pathological gait patterns and deviations (Winters et al., 1987; Rodda and Graham, 2001) may also present altered kinetic strategies (Steele et al., 2013) and should therefore be investigated further in addition to toe-walking.

The modeling framework developed in this study was based on 3-dimensional optical motion-capture gait data and an accurate 3-dimensional geometrical representation of the lower-limb based on cadaveric data (Carbone et al., 2015; De Pieri et al., 2018). However, due to the reduced number of markers on the foot segment, the motions of the ankle joint complex were simplified by neglecting subtalar inversion and eversion, which could affect the prediction of muscle activations (Jinha et al., 2006). Additionally, bone morphology variability in the transversal and frontal planes could also affect the prediction of muscle activations and forces (Passmore et al., 2018; Kainz et al., 2020; Martelli et al., 2020; De Pieri et al., 2021; Modenese et al., 2021; Veerkamp et al., 2021).

The lower-limb muscles were modeled as Hill-type actuators (Heinen et al., 2016) characterized by a muscle strength that followed force-length and force-velocity relationships (Hill, 1938; Zajac, 1989; Heinen et al., 2016; Andersen, 2021). Previous work underlined the importance of accounting for force-length relationships as a result of non-optimal contractile conditions associated with altered joint positions when toe-walking (Neptune et al., 2007). Specifically, the ankle plantarflexors are shortened during toe-walking and present, therefore, a reduced force-generating capacity (Herman and Bragin, 1967; Hoy et al., 1990; Perry et al., 2003). Patients with spasticity, prolonged equinus postures, and/or fixed muscular contractures may also present alteration of the underlying muscle morphology and intrinsic mechanical properties (Tardieu et al., 1982; Delp, 2003; Weidensteiner et al., 2021), even though it was shown that children with diplegic CP were able to generate maximum ankle torques at similar joint angles (Engsberg et al., 2000; Neptune et al., 2007). Therefore, restoring a more optimal foot and ankle position, as during heel-to-toe gait, may lead to improvements in muscle functionality and strength. On the other hand, muscle weakness is a commonly observed symptom in pathological populations (Hanssen et al., 2021) which can lead to inefficient and compensatory activations of other muscles, thus increasing the overall energetic cost of walking (van der Krogt et al., 2012). While the muscle strength of the participants in this study could be considered unimpaired, accounting for subject-specific scaling of muscle strength and mechanical properties may play a more important role in pathological populations (Kainz et al., 2018; Vandekerckhove et al., 2021).

The definition of the support moment as the weighted sum of the lower-limb joints sagittal extensive moments was derived from the mechanical interpretation provided by A.L. Hof (Hof, 2000) rather than the original formulation by D. Winter (Winter, 1980). According to this definition, the support moment counteracts the moment generated by the vertical component of the GRF times knee eccentricity and thus prevents the collapse of the knee (Hof, 2000). The comparison between the support moment and the vertical GRF moment showed good agreement for both walking modalities, with a similar overall trend and statistically significant differences limited to heel strike and pre-swing. This strengthens the interpretation that the support moment counteracts the external forces that would flex the knee. A higher support moment during toe-walking compared to normal walking indicates an overall higher muscular effort required when walking on the toes, as suggested by previous EMG analyses (Rose et al., 1999; Perry et al., 2003). It has to be mentioned that Eqs 2, 3 was derived under the approximation of equal shank and thigh lengths and while assuming massless legs (Hof, 2000). Neglecting the inertial contributions to the joint moments might therefore have affected the accuracy of the identity between the support moment and the moment produced by the external force. For more dynamic motions, such as running and jumping, this model might no longer be valid (Hof, 2000). The self-selected walking speed and the foot-floor inclination angle may further influence the resulting balance of forces and the predicted muscle contributions. This analysis was limited to the sagittal plane. Frontal plane balance and mediolateral stability are fundamental aspects of any support and locomotion strategy (Pandy et al., 2010). The lack of trunk markers did not allow to identify support strategies associated with upper-body control (Wyers et al., 2021). The control of the contralateral limb could also play an important role in forward propulsion while maintaining stability (Bovonsunthonchai et al., 2012). Nevertheless, the support moment can serve as a synthetic parameter to identify inefficient gait patterns and quantify the overall muscular demands associated with walking in pathological populations (Holsgaard-Larsen et al., 2019; Wyers et al., 2021).

The relationship between the progression moment $M_{P}$ , defined as the difference between hip and ankle extensive moments, and the moment generated by the transversal GRF component ( $F_{T}$ ) times the distance between hip and ankle ( p ) was also further evaluated. A good agreement between these two quantities was found for both walking modalities. This suggests that the forces acting transversally to the stance leg are counteracted by muscular contributions around the ankle and the hip, and not around the knee, as already suggested by Hof (2000). During toe-walking, altered activation of ankle and hip muscles is also required to maintain a force balance transversally to the leg axis. The relationship between these altered muscle activation patterns counteracting transversal forces during gait and different balance strategies, known from postural analyses, should be further evaluated (Horak and Nashner, 1986).

Muscle-induced acceleration analysis is another computational approach that can reveal how much each muscle supports the weight of the body and contributes to a forward acceleration during walking (Neptune et al., 2001; Neptune et al., 2004; Arnold et al., 2005; Liu et al., 2006; Sasaki et al., 2008; Hamner and Delp, 2013; Steele et al., 2013; Uchida and Delp, 2021). In addition to calculating the muscle-induced accelerations on the body center of mass, Neptune et al. (Neptune et al., 2001) computed the accelerations that the lower-limb muscles induce around the knee joint during walking. They observed that the vasti were the main contributors to knee stability during early stance while the rectus contributed towards terminal ST. The gastrocnemius induced the knee into flexion around mid-stance, while the soleus was the only muscle that provided knee stability throughout single-leg stance. These observations are in line with the muscle contributions to the support moment during normal walking presented in this study, further indicating that the support moment should be interpreted with a focus on knee stability rather than center of mass acceleration. The knee-extending effect of the triceps surae was previously explained through dynamic coupling (Zajac and Gordon, 1989; Uchida and Delp, 2021). This effect is clinically known as plantar flexion—knee extension couple (Gage, 1995; Brunner and Rutz, 2013; Sangeux et al., 2015). While the soleus causes an extension of the knee by accelerating the shank backwards (Anderson, 2006), the gastrocnemius can induce either a net knee flexion or extension, depending on ankle and knee positions (Zajac, 1993; Uchida and Delp, 2021). The analysis of the soleus contribution to the support moment confirmed an overall net knee-extensive activity for both walking modalities. On the other hand, the gastrocnemius worked as a net knee flexor during normal walking while it provided a small extensive contribution when walking on the toes. Neptune et al. (Neptune et al., 2001) further suggested that impaired soleus activity would require compensatory mechanisms to prevent the knee from collapsing, most probably through prolonged activation of the vasti, in agreement with clinical observations (Murray et al., 1978; Sutherland et al., 1980). The analysis of the muscle contributions to the individual joint moments and the support moment confirmed the important role of the vasti.

Nevertheless, the physical meaning of the muscle contributions determined through induced-acceleration analysis is not well-defined and should be interpreted carefully (Chen, 2006). The support and the progress moment definitions used in this study were also derived from simplified force equilibrium equations. While this study provides an intuitive interpretation of muscle forces as counteracting external forces to provide a net force and moment balance, conclusions about muscle functionality should also be drawn with caution in light of the previously-stated limitations. Further comparisons between support moment and induced-acceleration analyses of toe-walking are required to elucidate the physical meaning of the outcome measures of both analyses. Simulation frameworks able to predict *de novo* kinematics based on a mathematical model of the neuro-musculoskeletal system may overcome such limitations. These novel computational tools could better clarify muscle functionality by highlighting causal relationships between muscle characteristics or surgical intervention and the resulting overall motion pattern (Geijtenbeek, 2019; De Groote and Falisse, 2021; K.; Veerkamp et al., 2021).

The muscular demands and joint loads experienced during different motions and activities of daily living should also be the focus of further studies. In particular, sports activities that involve repetitive forefoot contact, such as running (Besier et al., 2009; Fredericson and Misra, 2012; Neal et al., 2016), jumping (Ferretti et al., 1983; Lian et al., 2017), or ballet (Prisk et al., 2008; Smith et al., 2015; Sobrino et al., 2015), may also lead to overuse injuries, such as patellofemoral pain syndrome, patellar tendonitis, Achilles tendonitis, and forefoot injuries. Similarly to toe-walking, the use of high-heeled shoes also leads to a plantarflexed position of the foot during walking, thus determining alterations of joint kinetics and muscle activations (Stefanyshyn et al., 2000; Simonsen et al., 2012).

## Conclusion

Musculoskeletal modeling allows investigating the kinetic requirements placed on individual muscles in association with a specific kinematic pattern, such as toe-walking. Walking on the toes, compared to a normal heel-to-toe gait pattern, induces altered kinetic balances at each joint level, thus determining functional adaptations for most lower-limb muscles, as shown in the analysis of a cohort of healthy volunteers mimicking a unilateral toe-walking pattern. Overall, the analysis of healthy individuals provides a unique insight on the muscular demands solely related to the specific gait pattern, independently of any underlying neuromuscular control disorder. It can be expected that similar activation patterns would be predicted through musculoskeletal modeling in both healthy and pathological subjects with similar gait patterns. Modeling predictions can be interpreted as the most optimal activation pattern required from the lower-limb muscles in order to walk in a specific manner. While a generalization of these conclusions to pathological populations should still be done cautiously, the method presented in this study has the potential to provide meaningful information for the clinical management of conditions characterized by altered gait patterns.

The support moment for instance can serve as a synthetic parameter to quantify the overall muscular demands associated with specific gait patterns. A higher support moment during toe-walking indicates an overall higher muscular effort necessary to maintain stance limb stability and prevent the collapse of the knee. Higher muscular demands during gait may lead to fatigue, pain, and reduced quality of life. Musculoskeletal modeling predictions suggest that toe-walking is indeed associated with significantly larger muscle forces exerted by the quadriceps to the patella, and prolonged force transmission through the Achilles tendon during the stance phase. The restoration of a normal heel-to-toe gait pattern may improve muscle functionality and gait efficiency, while reducing potential long-term adverse consequences, such as knee extensor overload. Optimal treatment options should therefore account for muscular demands and potential overloads associated with specific compensatory mechanisms.

## Data Availability

The raw data supporting the conclusion of this article will be made available by the authors without undue reservation.

## References

[B1] Alriksson‐Schmidt A. Hägglund G. (2016). Pain in Children and Adolescents with Cerebral Palsy: a Population‐based Registry Study. Acta Paediatr. 105 (6), 665–670. 10.1111/APA.13368 26880375 PMC5071732

[B2] Andersen M. S. Damsgaard M. Rasmussen J. (2009). Kinematic Analysis of Over-determinate Biomechanical Systems. Comp. Methods Biomech. Biomed. Eng. 12 (4), 371–384. 10.1080/10255840802459412 18949590

[B3] Andersen M. S. (2021). “Introduction to Musculoskeletal Modelling,” in Computational Modelling of Biomechanics and Biotribology in the Musculoskeletal System (Elsevier), 41–80. 10.1016/b978-0-12-819531-4.00004-3

[B4] Anderson F. (2006). “Simulation of Walking,” in Human Walking (Lippincott Williams & Wilkins), 195–210.

[B5] Arnold A. S. Anderson F. C. Pandy M. G. Delp S. L. (2005). Muscular Contributions to Hip and Knee Extension during the Single Limb Stance Phase of normal Gait: a Framework for Investigating the Causes of Crouch Gait. J. Biomech. 38 (11), 2181–2189. 10.1016/J.JBIOMECH.2004.09.036 16154404

[B6] Arnold A. S. Thelen D. G. Schwartz M. H. Anderson F. C. Delp S. L. (2007). Muscular Coordination of Knee Motion during the Terminal-Swing Phase of normal Gait. J. Biomech. 40 (15), 3314–3324. 10.1016/J.JBIOMECH.2007.05.006 17572431 PMC2795578

[B7] Balaban B. Tok F. (2014). Gait Disturbances in Patients with Stroke. PM&R 6 (7), 635–642. 10.1016/J.PMRJ.2013.12.017 24451335

[B8] Balemans A. C. J. van Wely L. Becher J. G. Dallmeijer A. J. (2015). Longitudinal Relationship Among Physical Fitness, Walking-Related Physical Activity, and Fatigue in Children with Cerebral Palsy. Oxford Acad. 95 (7), 996–1005. 10.2522/PTJ.20140270 25655878

[B9] Bertsch C. Unger H. Winkelmann W. Rosenbaum D. (2004). Evaluation of Early Walking Patterns from Plantar Pressure Distribution Measurements. First Year Results of 42 childrenGait & Posture. Gait & Posture 19 (3), 235–242. 10.1016/S0966-6362(03)00064-X 15125912

[B10] Besier T. F. Fredericson M. Gold G. E. Beaupré G. S. Delp S. L. (2009). Knee Muscle Forces during Walking and Running in Patellofemoral Pain Patients and Pain-free Controls. J. Biomech. 42 (7), 898–905. 10.1016/J.JBIOMECH.2009.01.032 19268945 PMC2671570

[B11] Booth A. T. C. Buizer A. I. Meyns P. Oude Lansink I. L. B. Steenbrink F. van der Krogt M. M. (2018). The Efficacy of Functional Gait Training in Children and Young Adults with Cerebral Palsy: a Systematic Review and Meta-Analysis. Dev. Med. Child. Neurol. 60 (9), 866–883. 10.1111/DMCN.13708 29512110

[B12] Boudarham J. Roche N. Pradon D. Bonnyaud C. Bensmail D. Zory R. (2013). Variations in Kinematics during Clinical Gait Analysis in Stroke Patients. PLoS ONE 8 (6), e66421. 10.1371/JOURNAL.PONE.0066421 23799100 PMC3684591

[B13] Bovonsunthonchai S. Hiengkaew V. Vachalathiti R. Vongsirinavarat M. Tretriluxana J. (2012). Effect of Speed on the Upper and Contralateral Lower Limb Coordination during Gait in Individuals with Stroke. Kaohsiung J. Med. Sci. 28 (12), 667–672. 10.1016/J.KJMS.2012.04.036 23217359 PMC11916217

[B14] Brunner R. Rutz E. (2013). Biomechanics and Muscle Function during Gait. J. Children's Orthopaedics 7 (5), 367–371. 10.1007/s11832-013-0508-510.1007/S11832-013-0508-5 PMC3838524 24432096

[B15] Brunner R. Taylor W. R. Visscher R. M. S. (2021). Restoration of Heel-Toe Gait Patterns for the Prevention of Asymmetrical Hip Internal Rotation in Patients with Unilateral Spastic Cerebral Palsy. Children 88 (9), 773773. 10.3390/CHILDREN8090773 PMC8467232 34572205

[B16] Carbone V. Fluit R. Pellikaan P. van der Krogt M. M. Janssen D. Damsgaard M. (2015). TLEM 2.0 - A Comprehensive Musculoskeletal Geometry Dataset for Subject-specific Modeling of Lower Extremity. J. Biomech. 48 (5), 734–741. 10.1016/j.jbiomech.2014.12.034 25627871

[B17] Chen G. (2006). Induced Acceleration Contributions to Locomotion Dynamics Are Not Physically Well Defined. Gait & Posture 23 (1), 37–44. 10.1016/J.GAITPOST.2004.11.016 16311193

[B18] Crowninshield R. D. Brand R. A. (1981). A Physiologically Based Criterion of Muscle Force Prediction in Locomotion. J. Biomech. 14 (11), 793–801. 10.1016/0021-9290(81)90035-X 7334039

[B19] Damiano D. L. Abel M. F. (1998). Functional Outcomes of Strength Training in Spastic Cerebral Palsy. Arch. Phys. Med. Rehabil. 79 (2), 119–125. 10.1016/S0003-9993(98)90287-8 9473991

[B20] Damsgaard M. Rasmussen J. Christensen S. T. Surma E. de Zee M. (2006). Analysis of Musculoskeletal Systems in the AnyBody Modeling System. Simulation Model. Pract. Theor. 14 (8), 1100–1111. 10.1016/j.simpat.2006.09.001

[B21] Davids J. R. Foti T. Dabelstein J. Bagley A. (1999). Voluntary (Normal) versus Obligatory (Cerebral Palsy) Toe-Walking in Children: A Kinematic, Kinetic, and Electromyographic Analysis. J. Pediatr. Orthopaedics 19 (4), 461–469. 10.1097/01241398-199907000-00008 10412994

[B22] De Groote F. Falisse A. (2021). Perspective on Musculoskeletal Modelling and Predictive Simulations of Human Movement to Assess the Neuromechanics of Gait. Proc. R. Soc. B. 288. 10.1098/RSPB.2020.2432 PMC7935082 33653141

[B23] De Pieri E. Friesenbichler B. List R. Monn S. Casartelli N. C. Leunig M. (2021). Subject-Specific Modeling of Femoral Torsion Influences the Prediction of Hip Loading during Gait in Asymptomatic Adults. Front. Bioeng. Biotechnol. 9, 551. 10.3389/fbioe.2021.679360 PMC8334869 34368092

[B24] De Pieri E. Lund M. E. Gopalakrishnan A. Rasmussen K. P. Lunn D. E. Ferguson S. J. (2018). Refining Muscle Geometry and Wrapping in the TLEM 2 Model for Improved Hip Contact Force Prediction. PLoS ONE 13 (9), e0204109. 10.1371/journal.pone.0204109 30222777 PMC6141086

[B25] De Pieri E. Lunn D. E. Chapman G. J. Rasmussen K. P. Ferguson S. J. Redmond A. C. (2019). Patient Characteristics Affect Hip Contact Forces during Gait. Osteoarthritis and Cartilage 27 (6), 895–905. 10.1016/j.joca.2019.01.016 30772383

[B26] Dean J. C. Kautz S. A. (2015). Foot Placement Control and Gait Instability Among People with Stroke. J. Rehabil. Res. Dev. 52 (5), 577–590. 10.1682/JRRD.2014.09.0207 26437301 PMC4737555

[B27] Delp S. L. (2003). What Causes Increased Muscle Stiffness in Cerebral Palsy? 27. United States: Muscle & nerve, 131–132. 10.1002/mus.10284 12548519

[B28] Derrick T. R. van den Bogert A. J. Cereatti A. Dumas R. Fantozzi S. Leardini A. (2020). ISB Recommendations on the Reporting of Intersegmental Forces and Moments during Human Motion Analysis. J. Biomech. 99, 109533. 10.1016/j.jbiomech.2019.109533 31791632

[B29] Eken M. M. Braendvik S. M. Bardal E. M. Houdijk H. Dallmeijer A. J. Roeleveld K. (2019). Lower Limb Muscle Fatigue during Walking in Children with Cerebral Palsy. Dev. Med. Child. Neurol. 61 (2), 212–218. 10.1111/DMCN.14002 30156008 PMC7379556

[B30] Engelbert R. Gorter J. W. Uiterwaal C. van de Putte E. Helders P. (2011). Idiopathic Toe-Walking in Children, Adolescents and Young Adults: a Matter of Local or Generalised Stiffness? BMC Musculoskelet. Disord. 12 (1), 1–8. 10.1186/1471-2474-12-61 21418634 PMC3070692

[B31] Engsberg J. R. Ross S. A. Olree K. S. Park T. S. (2000). Ankle Spasticity and Strength in Children with Spastic Diplegic Cerebral Palsy. Dev. Med. Child Neurol. 42 (1), 42–47. 10.1111/J.1469-8749.2000.TB00023.X 10665974

[B32] Erdemir A. McLean S. Herzog W. van den Bogert A. J. (2007). Model-based Estimation of Muscle Forces Exerted during Movements. Clin. Biomech. 22 (2), 131–154. 10.1016/j.clinbiomech.2006.09.005 17070969

[B33] Ferretti A. Ippolito E. Mariani P. Puddu G. (1983). Jumper's Knee. Am. J. Sports Med. 11 (2), 58–62. 10.1177/036354658301100202 6846682

[B34] Fock J. Galea M. P. Stillman B. C. Rawicki B. Clark M. (2009). Functional Outcome Following Botulinum Toxin A Injection to Reduce Spastic Equinus in Adults with Traumatic Brain Injury. Brain Inj. 18 (1), 57–63. 10.1080/0269905031000149498 14660236

[B35] Fredericson M. Misra A. K. (2007). Epidemiology and Aetiology of Marathon Running Injuries. Sports MedicineSpringer 3737 (4), 4437–4439. 10.2165/00007256-200737040-00043 17465629

[B36] Friston K. J. Holmes A. P. Worsley K. J. Poline J.-P. Frith C. D. Frackowiak R. S. J. (1994). Statistical Parametric Maps in Functional Imaging: A General Linear Approach. Hum. Brain Mapp. 2 (4), 189–210. 10.1002/hbm.460020402

[B37] Gage J. R. (1995). The Clinical Use of Kinetics for Evaluation of Pathologic Gait in Cerebral Palsy. Instr. Course Lect 44, 507–515. 7797889

[B38] Geijtenbeek T. (2019). SCONE: Open Source Software for Predictive Simulation of Biological Motion. Joss 4 (38), 1421. 10.21105/JOSS.01421

[B39] Graham H. K. Rosenbaum P. Paneth N. Dan B. Lin J.-P. Damiano D. L. (2016). Cerebral Palsy. Nat. Rev. Dis. Primers 2, 1–25. 10.1038/nrdp.2015.82 PMC9619297 27188686

[B40] Hallemans A. De Clercq D. Dongen S. V. Aerts P. (2006). Changes in Foot-Function Parameters during the First 5 Months after the Onset of Independent Walking: a Longitudinal Follow-Up Study. Gait & Posture 23 (2), 142–148. 10.1016/J.GAITPOST.2005.01.003 16399509

[B41] Hamner S. R. Delp S. L. (2013). Muscle Contributions to Fore-Aft and Vertical Body Mass center Accelerations over a Range of Running Speeds. J. Biomech. 46 (4), 780–787. 10.1016/J.JBIOMECH.2012.11.024 23246045 PMC3979434

[B42] Hanssen B. Peeters N. Vandekerckhove I. De Beukelaer N. Bar-On L. Molenaers G. (2021). The Contribution of Decreased Muscle Size to Muscle Weakness in Children with Spastic Cerebral Palsy. Front. Neurol. 12, 692582. 10.3389/FNEUR.2021.692582 34381414 PMC8350776

[B43] Heinen F. Lund M. E. Rasmussen J. de Zee M. (2016)., 230. London, England, 976–984. 10.1177/095441191665989410.1177/0954411916659894 Muscle-tendon Unit Scaling Methods of Hill-type Musculoskeletal Models: An Overview Proc. Inst. Mech. Eng. H 10 27459500

[B44] Herman R. Bragin S. J. (1967). Function of the Gastrocnemius and Soleus Muscles: A Preliminary Study in the Normal Human Subject. Oxford Acad. 47 (2), 105–113. 10.1093/PTJ/47.2.105 6045280

[B45] Hill A. V. (1938). The Heat of Shortening and the Dynamic Constants of Muscle. Ser. B-Biological Sci. 126 (843), 136–195.

[B46] Hof A. L. (2000). On the Interpretation of the Support Moment. Gait & Posture 12 (3), 196–199. 10.1016/S0966-6362(00)00084-9 11154929

[B47] Holsgaard-Larsen A. Thorlund J. B. Blackmore T. Creaby M. W. (2019). Changes in Total Lower Limb Support Moment in Middle-Aged Patients Undergoing Arthroscopic Partial Meniscectomy - A Longitudinal Observational Cohort Study. The Knee 26 (3), 595–602. 10.1016/J.KNEE.2019.04.004 31031126

[B48] Horak F. B. Nashner L. M. (1986). Central Programming of Postural Movements: Adaptation to Altered Support-Surface Configurations. J. Neurophysiol. 55 (6), 1369–1381. 10.1152/jn.1986.55.6.136910.1152/JN.1986.55.6.1369 3734861

[B49] Hoy M. G. Zajac F. E. Gordon M. E. (1990). A Musculoskeletal Model of the Human Lower Extremity: The Effect of Muscle, Tendon, and Moment Arm on the Moment-Angle Relationship of Musculotendon Actuators at the Hip, Knee, and Ankle. J. Biomech. 23 (2), 157–169. 10.1016/0021-9290(90)90349-8 2312520

[B50] Ikeda A. J. Abel M. F. Granata K. P. Damiano D. L. (1998). Quantification of Cocontraction in Spastic Cerebral Palsy. Electromyogr. Clin. Neurophysiol. 38 (8), 497–504. 9842485

[B51] Imani Nejad Z. Khalili K. Hosseini Nasab S. H. Schütz P. Damm P. Trepczynski A. (2020). The Capacity of Generic Musculoskeletal Simulations to Predict Knee Joint Loading Using the CAMS-Knee Datasets. Ann. Biomed. Eng. 48 (4), 1430–1440. 10.1007/S10439-020-02465-5/FIGURES/6 32002734 PMC7089909

[B52] Jahnsen R. Villien L. Aamodt G. Stanghelle J. Holm I. (2004). Musculoskeletal Pain in Adults with Cerebral Palsy Compared with the General Population. J. Rehabil. Med. 36 (2), 78–84. 10.1080/16501970310018305 15180222

[B53] Jinha A. Ait-Haddou R. Herzog W. (2006). Predictions of Co-contraction Depend Critically on Degrees-Of-freedom in the Musculoskeletal Model. J. Biomech. 39 (6), 1145–1152. 10.1016/J.JBIOMECH.2005.03.001 16549102

[B54] Kadaba M. P. Ramakrishnan H. K. Wootten M. E. (1990). Measurement of Lower Extremity Kinematics during Level Walking. J. Orthop. Res. 8 (3), 383–392. 10.1002/jor.1100080310 2324857

[B55] Kainz H. Goudriaan M. Falisse A. Huenaerts C. Desloovere K. De Groote F. (2018). The Influence of Maximum Isometric Muscle Force Scaling on Estimated Muscle Forces from Musculoskeletal Models of Children with Cerebral Palsy. Gait & Posture 65, 213–220. 10.1016/J.GAITPOST.2018.07.172 30558934

[B56] Kainz H. Killen B. A. Wesseling M. Perez-Boerema F. Pitto L. Garcia Aznar J. M. (2020). A Multi-Scale Modelling Framework Combining Musculoskeletal Rigid-Body Simulations with Adaptive Finite Element Analyses, to Evaluate the Impact of Femoral Geometry on Hip Joint Contact Forces and Femoral Bone Growth. PLoS ONE 15 (7 July), e0235966. 10.1371/journal.pone.0235966 32702015 PMC7377390

[B57] Kalen V. Adler N. Bleck E. E. (1986). Electromyography of Idiopathic Toe Walking. J. Pediatr. Orthopaedics 6 (1), 31–33. 10.1097/01241398-198601000-00006 3941176

[B58] Kelly I. P. Jenkinson A. Stephens M. O'Brien T. (1997). The Kinematic Patterns of Toe-Walkers. J. Pediatr. Orthopaedics 17 (4), 478–480. 10.1097/01241398-199707000-00013 9364387

[B59] Kerrigan D. C. Riley P. O. Rogan S. Burke D. T. (2000). Compensatory Advantages of Toe Walking. Arch. Phys. Med. Rehabil. 81 (1), 38–44. 10.1016/S0003-9993(00)90219-3 10638874

[B60] Kinsella S. Moran K. (2008). Gait Pattern Categorization of Stroke Participants with Equinus Deformity of the Foot. Gait & Posture 27 (1), 144–151. 10.1016/J.GAITPOST.2007.03.008 17467274

[B61] Lian Ø. B. Engebretsen L. Bahr R. (2017). Prevalence of Jumper's Knee Among Elite Athletes from Different Sports: A Cross-Sectional Study. Am. J. Sports Med. 33 (4), 561–567. 10.1177/036354650427045410.1177/0363546504270454 15722279

[B62] Liu M. Q. Anderson F. C. Pandy M. G. Delp S. L. (2006). Muscles that Support the Body Also Modulate Forward Progression during Walking. J. Biomech. 39 (14), 2623–2630. 10.1016/J.JBIOMECH.2005.08.017 16216251

[B63] Lund M. E. Andersen M. S. de Zee M. Rasmussen J. (2015). Scaling of Musculoskeletal Models from Static and Dynamic Trials. Int. Biomech. 2 (1), 1–11. 10.1080/23335432.2014.993706

[B64] Lund M. E. de Zee M. Andersen M. S. Rasmussen J. (2012). On Validation of Multibody Musculoskeletal Models. Proc. Inst. Mech. Eng. H 226 (2), 82–94. 10.1177/0954411911431516 22468460

[B65] Lund M. Rasmussen J. Andersen M. (2019). AnyPyTools: A Python Package for Reproducible Research with the AnyBody Modeling System. Joss 4 (33), 1108. 10.21105/joss.01108

[B66] Lundh S. Nasic S. Riad J. (2018). Fatigue, Quality of Life and Walking Ability in Adults with Cerebral Palsy. Gait & Posture 61, 1–6. 10.1016/J.GAITPOST.2017.12.017 29277025

[B67] Lunn D. E. De Pieri E. Chapman G. J. Lund M. E. Redmond A. C. Ferguson S. J. (2020). Current Preclinical Testing of New Hip Arthroplasty Technologies Does Not Reflect Real-World Loadings: Capturing Patient-specific and Activity-Related Variation in Hip Contact Forces. The J. Arthroplasty 35 (3), 877–885. 10.1016/j.arth.2019.10.006 31699529

[B68] Manca M. Ferraresi G. Cosma M. Cavazzuti L. Morelli M. Benedetti M. G. (20142014). “Gait Patterns in Hemiplegic Patients with Equinus Foot Deformity,” in BioMed Research International. Editor Morone G., 2014, 1–7. 10.1155/2014/939316 PMC4016931 24967417

[B69] Marra M. A. Vanheule V. Fluit R. Koopman B. H. F. J. M. Rasmussen J. Verdonschot N. (2015). A Subject-specific Musculoskeletal Modeling Framework to Predict *In Vivo* Mechanics of Total Knee Arthroplasty. ASME 137 (2), 020904. 10.1115/1.4029258 25429519

[B70] Martelli S. Sancisi N. Conconi M. Pandy M. G. Kersh M. E. Parenti-Castelli V. (2020). The Relationship between Tibiofemoral Geometry and Musculoskeletal Function during normal Activity. Gait & Posture 80, 374–382. 10.1016/J.GAITPOST.2020.06.022 32622207

[B71] McGinley J. L. Dobson F. Ganeshalingam R. Shore B. J. Rutz E. Graham H. K. (2012). Single-event Multilevel Surgery for Children with Cerebral Palsy: a Systematic Review. Dev. Med. Child. Neurol. 54 (2), 117–128. 10.1111/J.1469-8749.2011.04143.X 22111994

[B72] Modenese L. Barzan M. Carty C. P. (2021). Dependency of Lower Limb Joint Reaction Forces on Femoral Version. Gait & Posture 88, 318–321. 10.1016/J.GAITPOST.2021.06.014 34246172

[B73] Morgan P. McGinley J. (2013). Gait Function and Decline in Adults with Cerebral Palsy: a Systematic Review. Disabil. Rehabil. 36 (1), 1–9. 10.3109/09638288.2013.77535910.3109/09638288.2013.775359 23594053

[B74] Mortensen J. Trkov M. Merryweather A. (2018). Exploring Novel Objective Functions for Simulating Muscle Coactivation in the Neck. J. Biomech. 71, 127–134. 10.1016/J.JBIOMECH.2018.01.030 29452757

[B75] Murray M. P. Guten G. N. Sepic S. B. Gardner G. M. Baldwin J. M. (1978). Function of the Triceps Surae during Gait. Compensatory Mechanisms for Unilateral Loss. The J. Bone Jt. SurgeryAmerican volume 60 (4), 473–476. 10.2106/00004623-197860040-00007 670268

[B76] Neal B. S. Barton C. J. Gallie R. O’Halloran P. Morrissey D. (2016). Runners with Patellofemoral Pain Have Altered Biomechanics Which Targeted Interventions Can Modify: A Systematic Review and Meta-Analysis. Gait & Posture 45, 69–82. 10.1016/J.GAITPOST.2015.11.018 26979886

[B77] Neptune R. R. Burnfield J. M. Mulroy S. J. (2007). The Neuromuscular Demands of Toe Walking: A Forward Dynamics Simulation Analysis. J. Biomech. 40 (6), 1293–1300. 10.1016/J.JBIOMECH.2006.05.022 16842801

[B78] Neptune R. R. Kautz S. A. Zajac F. E. (2001). Contributions of the Individual Ankle Plantar Flexors to Support, Forward Progression and Swing Initiation during Walking. J. Biomech. 34 (11), 1387–1398. 10.1016/S0021-9290(01)00105-1 11672713

[B79] Neptune R. R. Zajac F. E. Kautz S. A. (2004). Muscle Force Redistributes Segmental Power for Body Progression during Walking. Gait & Posture 19 (2), 194–205. 10.1016/S0966-6362(03)00062-6 15013508

[B80] Opheim A. Jahnsen R. Olsson E. Stanghelle J. K. (2009). Walking Function, Pain, and Fatigue in Adults with Cerebral Palsy: a 7-year Follow-Up Study. Dev. Med. Child Neurol. 51 (5), 381–388. 10.1111/J.1469-8749.2008.03250.X 19207296

[B81] Palmer F. B. Shapiro B. K. Wachtel R. C. Allen M. C. Hiller J. E. Harryman S. E. (2010). The Effects of Physical Therapy on Cerebral Palsy. A Controlled Trial in Infants with Spastic Diplegia. N. Engl. J. Med. 318 (13), 803–808. 10.1056/NEJM19880331318130210.1056/NEJM198803313181302 3280999

[B82] Pandy M. G. Lin Y.-C. Kim H. J. (2010). Muscle Coordination of Mediolateral Balance in normal Walking. J. Biomech. 43 (11), 2055–2064. 10.1016/J.JBIOMECH.2010.04.010 20451911

[B83] Passmore E. Graham H. K. Pandy M. G. Sangeux M. (2018). Hip- and Patellofemoral-Joint Loading during Gait Are Increased in Children with Idiopathic Torsional Deformities. Gait & Posture 63, 228–235. 10.1016/j.gaitpost.2018.05.003 29775910

[B84] Pataky T. C. (2012). One-dimensional Statistical Parametric Mapping in Python. Comp. Methods Biomech. Biomed. Eng. 15 (3), 295–301. 10.1080/10255842.2010.527837 21756121

[B85] Pataky T. C. Vanrenterghem J. Robinson M. A. (2015). Zero- vs. One-Dimensional, Parametric vs. Non-parametric, and Confidence Interval vs. Hypothesis Testing Procedures in One-Dimensional Biomechanical Trajectory Analysis. J. Biomech. 48 (7), 1277–1285. 10.1016/J.JBIOMECH.2015.02.051 25817475

[B86] Perry J. Burnfield J. M. Gronley J. K. Mulroy S. J. (2003). Toe Walking: Muscular Demands at the Ankle and Knee. Arch. Phys. Med. Rehabil. 84 (1), 7–16. 10.1053/APMR.2003.50057 12589614

[B87] Prisk V. R. O'Loughlin P. F. Kennedy J. G. (2008). Forefoot Injuries in Dancers. Clin. Sports Med. 27 (2), 305–320. 10.1016/J.CSM.2007.12.005 18346545

[B88] Rasmussen J. Damsgaard M. Voigt M. (2001). Muscle Recruitment by the Min/max Criterion - a Comparative Numerical Study. J. Biomech. 34 (3), 409–415. 10.1016/S0021-9290(00)00191-3 11182135

[B89] Rethlefsen S. A. Nguyen D. T. Wren T. A. L. Milewski M. D. Kay R. M. (2015). Knee Pain and Patellofemoral Symptoms in Patients with Cerebral Palsy. J. Pediatr. Orthopaedics 35 (5), 519–522. 10.1097/BPO.0000000000000304 25171680

[B90] Rodda J. Graham H. K. (2001). Classification of Gait Patterns in Spastic Hemiplegia and Spastic Diplegia: A Basis for a Management Algorithm. Eur. J. Neurol. 8 (s5), 98–108. 10.1046/j.1468-1331.2001.00042.x 11851738

[B91] Romkes J. Brunner R. (2007). An Electromyographic Analysis of Obligatory (Hemiplegic Cerebral Palsy) and Voluntary (normal) Unilateral Toe-Walking. Gait & Posture 26 (4), 577–586. 10.1016/J.GAITPOST.2006.12.010 17275305

[B92] Rose J. Martin J. G. Torburn L. Rinsky L. A. Gamble J. G. (1999). Electromyographic Differentiation of Diplegic Cerebral Palsy from Idiopathic Toe Walking: Involuntary Coactivation of the Quadriceps and Gastrocnemius. J. Pediatr. Orthopaedics 19 (5), 677–682. 10.1097/01241398-199909000-00025 10488875

[B93] Sangeux M. Rodda J. Graham H. K. (2015). Sagittal Gait Patterns in Cerebral Palsy: The Plantarflexor-Knee Extension Couple index. Gait & Posture 41 (2), 586–591. 10.1016/J.GAITPOST.2014.12.019 25604121

[B94] Sasaki K. Neptune R. R. Burnfield J. M. Mulroy S. J. (2008). Muscle Compensatory Mechanisms during Able-Bodied Toe Walking. Gait & Posture 27 (3), 440–446. 10.1016/J.GAITPOST.2007.05.012 17624784

[B95] Schlough K. Andre K. Owen M. Adelstein L. Hartford M. C. Javier B. (2020). Differentiating between Idiopathic Toe Walking and Cerebral Palsy: A Systematic Review. Lippincott Williams and Wilkins 32 (1), 2–10. 10.1097/PEP.0000000000000659 31842091

[B96] Schwachmeyer V. Damm P. Bender A. Dymke J. Graichen F. Bergmann G. (2013). *In Vivo* hip Joint Loading during post-operative Physiotherapeutic Exercises. PloS one 8 (10), e77807. 10.1371/journal.pone.0077807 24204977 PMC3812157

[B97] Schweizer K. Romkes J. Brunner R. (2013). The Association between Premature Plantarflexor Muscle Activity, Muscle Strength, and Equinus Gait in Patients with Various Pathologies. Res. Dev. Disabilities 34 (9), 2676–2683. 10.1016/J.RIDD.2013.05.025 23764825

[B98] Seth A. Hicks J. L. Uchida T. K. Habib A. Dembia C. L. Dunne J. J. (2018). OpenSim: Simulating Musculoskeletal Dynamics and Neuromuscular Control to Study Human and Animal Movement. Plos Comput. Biol. 14 (7), e1006223. 10.1371/JOURNAL.PCBI.1006223 30048444 PMC6061994

[B99] Simonsen E. B. Svendsen M. B. Nørreslet A. Baldvinsson H. K. Heilskov-Hansen T. Larsen P. K. (2012). Walking on High Heels Changes Muscle Activity and the Dynamics of Human Walking Significantly. J. Appl. Biomech. 28 (1), 20–28. 10.1123/JAB.28.1.20 22431211

[B100] Sloot L. H. van der Krogt M. M. (2018). Interpreting Joint Moments and Powers in Gait BT - Handbook of Human Motion. Cham: Springer International Publishing, 625–643. 10.1007/978-3-319-14418-4_32

[B101] Smith P. J. Gerrie B. J. Varner K. E. McCulloch P. C. Lintner D. M. Harris J. D. (2015). Incidence and Prevalence of Musculoskeletal Injury in Ballet. Orthopaedic J. Sports Med. 3 (7), 232596711559262. 10.1177/232596711559262110.1177/2325967115592621 PMC4622328 26673541

[B102] Smith S. H. L. Coppack R. J. van den Bogert A. J. Bennett A. N. Bull A. M. J. (2021). Review of Musculoskeletal Modelling in a Clinical Setting: Current Use in Rehabilitation Design, Surgical Decision Making and Healthcare Interventions. Clin. Biomech. 83, 105292. 10.1016/J.CLINBIOMECH.2021.105292 33588135

[B103] Sobrino F. J. de la Cuadra C. Guillén P. (2015). Overuse Injuries in Professional Ballet. Orthopaedic J. Sports Med. 3 (6), 232596711559011. 10.1177/2325967115590114 PMC4622371 26665100

[B104] Solan M. C. Kohls-Gatzoulis J. Stephens M. M. (2010). Idiopathic Toe Walking and Contractures of the Triceps Surae. Foot Ankle Clin. 15 (2), 297–307. 10.1016/J.FCL.2010.01.002 20534357

[B105] Solomonow M. Baratta R. Zhou B. H. D'Ambrosia R. (1988). Electromyogram Coactivation Patterns of the Elbow Antagonist Muscles during Slow Isokinetic Movement. Exp. Neurol. 100 (3), 470–477. 10.1016/0014-4886(88)90032-5 3366200

[B106] Steele K. M. Rozumalski A. Schwartz M. H. (2015). Muscle Synergies and Complexity of Neuromuscular Control during Gait in Cerebral Palsy. Dev. Med. Child. Neurol. 57 (12), 1176–1182. 10.1111/dmcn.12826 26084733 PMC4683117

[B107] Steele K. M. Seth A. Hicks J. L. Schwartz M. H. Delp S. L. (2013). Muscle Contributions to Vertical and Fore-Aft Accelerations Are Altered in Subjects with Crouch Gait. Gait & Posture 38 (1), 86–91. 10.1016/J.GAITPOST.2012.10.019 23200083 PMC3600387

[B108] Stefanyshyn D. J. Nigg B. M. Fisher V. O'Flynn B. Liu W. (2000). The Influence of High Heeled Shoes on Kinematics, Kinetics, and Muscle EMG of Normal Female Gait. J. Appl. Biomech. 16 (3), 309–319. 10.1123/JAB.16.3.309

[B109] Strobl W. Theologis T. Brunner R. Kocer S. Viehweger E. Pascual-Pascual I. (2015). Best Clinical Practice in Botulinum Toxin Treatment for Children with Cerebral Palsy. Toxins 7 (5), 1629–1648. 10.3390/toxins7051629 25969944 PMC4448165

[B110] Sun D. Fekete G. Baker J. S. Mei Q. István B. Zhang Y. (20202020). A Pilot Study of Musculoskeletal Abnormalities in Patients in Recovery from a Unilateral Rupture-Repaired Achilles Tendon. Ijerph 1717 (13), 46424642. 10.3390/IJERPH17134642 PMC7369810 32605170

[B111] Sutherland D. H. Cooper L. Daniel D. (1980). The Role of the Ankle Plantar Flexors in normal Walking. J. Bone Jt. SurgeryAmerican volume 62 (3), 354–363. 10.2106/00004623-198062030-00005 7364808

[B112] Sylvester A. D. Lautzenheiser S. G. Kramer P. A. (2021). A Review of Musculoskeletal Modelling of Human Locomotion. Interf. Focus. 11 (5), 20200060. 10.1098/RSFS.2020.0060 PMC8361578 34938430

[B113] Tardieu C. Huet de la Tour E. Bret M. D. Tardieu G. (1982). Muscle Hypoextensibility in Children with Cerebral Palsy: I. Clinical and Experimental Observations. Arch. Phys. Med. Rehabil. 63 (3), 97–102. 7073456

[B114] Tardieu C. Lespargot A. Tabary C. Bret M.-D. (1989). Toe-Walking in Children with Cerebral Palsy: Contributions of Contracture and Excessive Contraction of Triceps Surae Muscle. Oxford Acad. 69 (8), 656–662. 10.1093/PTJ/69.8.656 2748720

[B115] Thelen D. G. Lenz A. Hernandez A. (2011). Measurement and Simulation of Joint Motion Induced via Biarticular Muscles during Human Walking. Proced. IUTAM 2, 290–296. 10.1016/J.PIUTAM.2011.04.026

[B116] Totah D. Menon M. Jones-Hershinow C. Barton K. Gates D. H. (2019). The Impact of Ankle-Foot Orthosis Stiffness on Gait: A Systematic Literature Review. Gait & Posture 69, 101–111. 10.1016/J.GAITPOST.2019.01.020 30708092

[B117] Uchida T. K. Delp S. L. (2021). Biomechanics of Movement: The Science of Sports, Robotics, and Rehabilitation. MIT Press.

[B118] Unnithan V. B. Dowling J. J. Frost G. Volpe Ayub B. Bar-Or O. (1996). Cocontraction and Phasic Activity during GAIT in Children with Cerebral Palsy. Electromyogr. Clin. Neurophysiol. 36 (8), 487–494. 8985677

[B119] van der Krogt M. M. Delp S. L. Schwartz M. H. (2012). How Robust Is Human Gait to Muscle Weakness? Gait & Posture 36 (1), 113–119. 10.1016/j.gaitpost.2012.01.017 22386624 PMC4890623

[B120] Vandekerckhove I. Wesseling M. Kainz H. Desloovere K. Jonkers I. (2021). The Effect of Hip Muscle Weakness and Femoral Bony Deformities on Gait Performance. Gait & Posture 83, 280–286. 10.1016/j.gaitpost.2020.10.022 33227606

[B121] Veerkamp K. Kainz H. Killen B. A. Jónasdóttir H. van der Krogt M. M. (2021). Torsion Tool: An Automated Tool for Personalising Femoral and Tibial Geometries in OpenSim Musculoskeletal Models. J. Biomech. 125, 110589. 10.1016/J.JBIOMECH.2021.110589 34218040

[B122] Veerkamp K. Schallig W. Harlaar J. Pizzolato C. Carty C. P. Lloyd D. G. (2019). The Effects of Electromyography-Assisted Modelling in Estimating Musculotendon Forces during Gait in Children with Cerebral Palsy. J. Biomech. 92, 45–53. 10.1016/j.jbiomech.2019.05.026 31153626

[B123] Veerkamp K. Waterval N. F. J. Geijtenbeek T. Carty C. P. Lloyd D. G. Harlaar J. (2021). Evaluating Cost Function Criteria in Predicting Healthy Gait. J. Biomech. 123, 110530. 10.1016/J.JBIOMECH.2021.110530 34034014

[B124] Vigotsky A. D. Zelik K. E. Lake J. Hinrichs R. N. (2019). Mechanical Misconceptions: Have We Lost the "mechanics" in "sports Biomechanics"? J. Biomech. 93, 1–5. 10.1016/J.JBIOMECH.2019.07.005 31337496

[B125] Villani C. Pappalardo S. Meloni C. Amorese V. Romanini L. (1988). Patellofemoral Dysplasia in Infantile Cerebral Palsy. Ital. J. Orthop. Traumatol. 14 (2), 201–210. 3220725

[B126] Weidensteiner C. Madoerin P. Deligianni X. Haas T. Bieri O. Akinci D'Antonoli T. (2021). Quantification and Monitoring of the Effect of Botulinum Toxin A on Paretic Calf Muscles of Children with Cerebral Palsy with MRI: A Preliminary Study. Front. Neurol. 12, 390. 10.3389/FNEUR.2021.630435 PMC8085320 33935939

[B127] Williams C. Haines T. (2015). Idiopathic Toe Walking May Impact on Quality of Life. J. Foot Ankle Res. 8 (Suppl. 2), O40. 10.1186/1757-1146-8-S2-O40

[B128] Williams G. Morris M. E. Schache A. McCrory P. R. (2009). Incidence of Gait Abnormalities after Traumatic Brain Injury. Arch. Phys. Med. Rehabil. 90 (4), 587–593. 10.1016/J.APMR.2008.10.013 19345773

[B129] Winter D. A. (2009). Biomechanics and Motor Control of Human Movement. John Wiley & Sons.

[B130] Winter D. A. (1980). Overall Principle of Lower Limb Support during Stance Phase of Gait. J. Biomech. 13 (11), 923–927. 10.1016/0021-9290(80)90162-1 7275999

[B131] Winters T. F. Gage J. R. Hicks R. (1987). Gait Patterns in Spastic Hemiplegia in Children and Young Adults. J. Bone Jt. Surg AmAmerican volume 69 (3), 437–441. 3818706

[B132] Wu G. Siegler S. Allard P. Kirtley C. Leardini A. Rosenbaum D. (2002). ISB Recommendation on Definitions of Joint Coordinate System of Various Joints for the Reporting of Human Joint Motion-Part I: Ankle, Hip, and Spine. J. Biomech. 35 (4), 543–548. 10.1016/S0021-9290(01)00222-6 11934426

[B133] Wyers L. Verheyen K. Ceulemans B. Schoonjans A.-S. Desloovere K. Van de Walle P. (2021). The Mechanics behind Gait Problems in Patients with Dravet Syndrome. Gait & Posture 84, 321–328. 10.1016/J.GAITPOST.2020.12.029 33445141

[B134] Zajac F. E. (1989). Muscle and Tendon: Properties, Models, Scaling, and Application to Biomechanics and Motor Control. Crit. Rev. Biomed. Eng. 17 (4), 359–411. 2676342

[B135] Zajac F. E. Gordon M. E. (1989). Determining Muscle??s Force and Action in Multi-Articular Movement. Exerc. Sport Sci. Rev. 16, 187–230. 10.1249/00003677-198900170-00009 2676547

[B136] Zajac F. E. (1993). Muscle Coordination of Movement: A Perspective. J. Biomech. 26 (Suppl. 1), 109–124. 10.1016/0021-9290(93)90083-Q 8505346

[B137] Zeininger A. D. Jensen J. L. Shapiro L. J. (2018). Ontogenetic Changes in Foot Strike Pattern and Calcaneal Loading during Walking in Young Children. Gait & Posture 59, 18–22. 10.1016/J.GAITPOST.2017.09.027 28982055

